# A bird’s-eye view of Italian genomic variation through whole-genome sequencing

**DOI:** 10.1038/s41431-019-0551-x

**Published:** 2019-11-29

**Authors:** Massimiliano Cocca, Caterina Barbieri, Maria Pina Concas, Antonietta Robino, Marco Brumat, Ilaria Gandin, Matteo Trudu, Cinzia Felicita Sala, Dragana Vuckovic, Giorgia Girotto, Giuseppe Matullo, Ozren Polasek, Ivana Kolčić, Paolo Gasparini, Nicole Soranzo, Daniela Toniolo, Massimo Mezzavilla

**Affiliations:** 1 Institute for Maternal and Child Health IRCCS Burlo Garofolo, Trieste, Italy; 2 Division of Genetics and Cell Biology, San Raffaele Scientific Institute, Milan, Italy; 3 Department of Medical, Surgical and Health Sciences, University of Trieste, Trieste, Italy; 4 Molecular Genetics of Renal Disorders Unit, Division of Genetics and Cell Biology, San Raffaele Scientific Institute, Milan, Italy; 5 Sanger Institute, Wellcome Trust Genome Campus, Hinxton, CB10 1SA UK; 6 Department of Medical Sciences, University of Turin, Turin, Italy; 7 Italian Institute for Genomic Medicine (IIGM), Turin, Italy; 8 Public Health, University of Split, Split, Croatia

**Keywords:** Rare variants, Next-generation sequencing

## Abstract

The genomic variation of the Italian peninsula populations is currently under characterised: the only Italian whole-genome reference is represented by the Tuscans from the 1000 Genome Project. To address this issue, we sequenced a total of 947 Italian samples from three different geographical areas. First, we defined a new Italian Genome Reference Panel (IGRP1.0) for imputation, which improved imputation accuracy, especially for rare variants, and we tested it by GWAS analysis on red blood traits. Furthermore, we extended the catalogue of genetic variation investigating the level of population structure, the pattern of natural selection, the distribution of deleterious variants and occurrence of human knockouts (HKOs). Overall the results demonstrate a high level of genomic differentiation between cohorts, different signatures of natural selection and a distinctive distribution of deleterious variants and HKOs, confirming the necessity of distinct genome references for the Italian population.

## Introduction

Large sequencing projects have identified the majority of common variants and millions of rare and low-frequency variants in populations of northern European ancestry [1–3]. Most of the rare variants were found in protein-coding genes. Moreover, it was calculated that each individual might carry more than 20,000 variants per exome [4, 5], a finding that makes even more challenging to understand gene function and the impact of each rare variant. From this point of view, the catalogue of rare and low-frequency variants is still mostly incomplete, and its completion will represent a significant challenge. A challenge that starts with the filtering of candidate variants by frequency in sequenced cohorts. The efficacy of such filtering depends on both the size and the genetic diversity of the available reference data. In the available human genome reference data (e.g. 1000G Phase 3, ExAC databases), Southern European populations - which represent a significant proportion of the overall European populations - are highly underrepresented (i.e. only a small group of subjects from Italy - Tuscany - and Spain). In particular, the Italian peninsula, characterised by past and recent migration events [6, 7] and also widespread isolation [8–10] is a fascinating region to describe and understand. We characterised whole-genome sequences from isolated populations localised in three different geographical areas of Italy: North-West (Val Borbera - VBI), North-East (Friuli Venezia Giulia - FVG) and South-East (Carlantino - CAR); for which the presence of stratification [8, 11] and the different levels of isolation were demonstrated [12]. These populations belong to the INGI (Italian Network of Genetic Isolates) network. In isolated populations, variants that are rare or absent elsewhere can occur at higher frequencies. In this respect, our Italian genomes could be extremely useful for the genetic analysis of other Italian and South-European populations, in a similar way as already shown in recent studies describing the advantages of WGS study-cohort based reference panels [1, 13–16]. With our study, we sought to answer the following questions: (1) Are we able to increment the catalogue of genotypic variation, possibly in the low-frequency spectrum, with new data? (2) Do we add useful information in terms of genetic variability, and are they non-redundant concerning the South-European-Italian data already present in the commonly used reference panels for imputation? (3) Will we be able to identify new loci/variants, characteristic of a South-European subpopulation through GWAS, using the new reference panel for imputation? (4) How homogeneous are genomes coming from different regions of Italy in terms of population structure, natural selection signatures, deleterious variants distribution and human knockouts (HKO)? Moreover, as a consequence, how reliable is to use only one reference population for Italians such as Tuscans?

## Materials and methods

### WGS data generation: variant calling and quality control

Samples were randomly selected for all cohorts. The sequencing was carried out at different sequencing centres: the Wellcome Trust Sanger Institute in Hinxton (UK), BGI, Shenzhen (PRC) and the San Raffaele Hospital (HSR) in Milan. Alignment to the Human genome reference build 37 (GRCh37) was performed with *bwa* software [17] and, each bam file was improved through multiple steps as detailed in Supplementary Notes. After extensive quality control of the raw data (Supplementary Notes), a total of 947 samples was sent forward for the Variant Calling step. We separately produced genotype calls for autosomal chromosomes for each population and annotated each resulting variant set with information provided by the Variant Effect Predictor tool v.90 [18]. A detailed description of the pipeline used is provided in Supplementary Notes. Samples and sites were investigated for outliers or artefacts after the variant calling (Supplementary Notes).

### Reference imputation panel

For each INGI cohort, we included SNPs and INDELs from WGS data in the reference panel according to the following criteria: (a) all sites with Alternative Allele count (AC) ≥ 2 and Read depth (DP) ≥ 5; (b) all sites with AC = 1 in each cohort, either shared at least between two INGI cohorts or shared with at least one of the external resources selected (UK10K and 1000G Project Phase 3). This last match was performed by comparing position, reference and alternative allele. The data were added to the 1000G Phase 3 reference panel, using the method implemented by the IMPUTE2 software [19], to obtain a final reference (Italian Genome Reference Panel v1.0, IGRP1.0 from now on). We performed the imputation test on chromosome 2 genotypes in different cohorts: (a) INGI cohorts; (b) a cohort of 567 unselected outbred samples from North Western Italy (NW-ITALY); (c) three cohorts from Croatia (VIS - 960 samples, KORCULA - 1812 samples and SPLIT - 466 samples). We compared the imputation metrics across the different panels for each population. We assessed the *r*^2^ metric, which estimates the correlation between the real genotype and the imputed genotype and the IMPUTE info score parameter, which provides a measure of the observed statistical information associated with the allele frequency estimate for each variant [19]. We removed from each INGI cohort all the samples represented in the reference panel.

### Genome-wide association studies (GWAS)

GWA studies on Red Blood traits (MCH - Mean Corpuscular Haemoglobin, HGB - Haemoglobin, MCHC - Mean Corpuscular Haemoglobin Concentration, RBC - Red Blood Cell count, HCT - Hematocrit, MCV - Mean Corpuscular Volume) were performed in each population separately, using age and gender as covariates in an additive model, once using 1000G imputation and once IGRP1.0. The analyses were performed using the mixed linear models as implemented in R ABEL packages [20]. We excluded variants with info score ≤ 0.4 if the MAF was ≥ 1%. For rare variants (MAF 0.1–1%), we used a more stringent Info Score cut-off ( ≥ 0.8) [13]. Meta-analysis was performed using the software METAL [21]. After meta-analysis, the variants that were not present with the same direction in at least two of the three cohorts were excluded [22, 23]. Bonferroni correction was applied. Genomic positions are referred to the GRCh37. Manhattan plots were generated with the R library qqman [24] and hudson package [25].

### Population structure

We carried out the Principal component analysis (PCA) to define the genetic structure of our population using PLINK [26]. PCA was carried out after removing markers in high LD (*r*^2^ > 0.4), using the function --indep-pairwise 200 50 0.4 and with MAF < 0.02, after filtering a total of 695,052 variants were used. Runs of homozygosity (ROH) and inbreeding coefficient were estimated as well using PLINK. More details are reported in Supplementary Notes. Pairwise Fst was calculated using the software 4p [27]. Tree graph analysis was performed using Treemix [28]. The analysis of ancestry components was done using ADMIXTURE v1.2 [29]. Cross-validation error procedure was implemented to select the best cluster solution. All the analyses were performed on a subset of 46 individuals form each subpopulations.

### Natural selection

We estimated the level of positive selection for each population using the iHS statistic [30] implemented in the *selscan* programme [31]. We used only markers with MAF > 0.05 in each population. Furthermore, we adopted a conservative approach for genes under putative positive selection: we selected only genes with at least 20 markers with standardised |iHS| ≥ 2. We created a second subset of genes selecting the ones with least 20 markers with standardised |iHS| ≥ 2.5.

### Deleterious variants

After the exclusion of multiallelic variants, we subdivided all variant in bins according to their CADD [32] score and frequency. The following AC classes were defined: between 1–2 AC, 3–5 AC, 5–10 AC and more than 10 AC; thus the variants were binned in the following CADD categories 0–5, 5–15,15–20 > 20. We then applied the DVxy statistic as described in Xue et al. [12]. using as reference the TSI population from 1000 Genomes. Also, we estimated the ratio of private and shared DV variants (variants enriched).

### Human knockouts

To identify HKO, we considered only deleterious variants in protein-coding genes: we firstly selected variants with high impact as defined by VEP (i.e. frameshift, splice acceptor variant, splice donor variant, stop gained, stop lost, start lost, transcript ablation, transcript amplification) and CADD score ≥ 20. We defined as putative HKO only those presenting at least one homozygous individual in one population. HKO’s were classified as TOTAL when the variant was predicted as LOF in all Ensembl database transcripts. Otherwise, they were classified as PARTIAL. After filtering for TOTAL HKO, the average number of HKO variants per individual was 20 (12–31), in agreement with the previous determinations [33]. Overlaps of HKOs between populations were analysed using the R package “VennDiagram” [34]. RVIS score for each gene was also collected [35].

## Results

### WGS data generation: variant calling and quality control

**Fig. 1:**
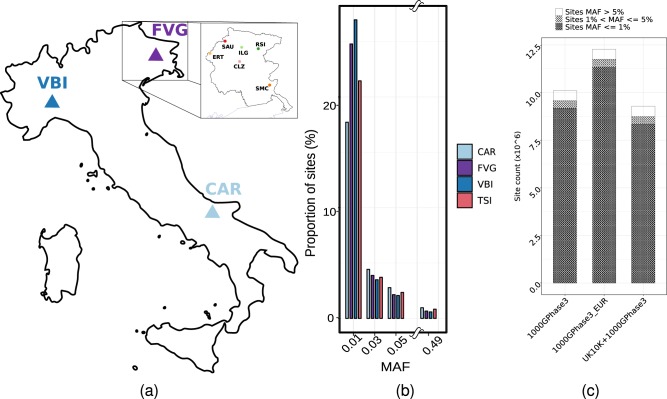
Dataset description: **a** Geographical localisation of the three study cohorts. **b** The minor allele frequency spectrum of the final INGI data set. For comparison, the Minor allele frequency spectrum of the TSI cohort from 1000G Phase 3 data has been added. **c** The stacked bar-plot represent the number of novel sites identified in the whole INGI dataset, compared with the available resources. The majority of the private INGI sites are in the range of the rare variants (MAF < = 1% - cross-pattern). Singletons sites (AC = 1) are included

A total of 926 samples passed all the quality control steps (Table 1). Approximately 27M sites (i.e. >24M SNVs and >2M small insertions and deletions, INDELs) were detected (Table 1) in the joint dataset. Overall, 7.1M sites (26%) were common (MAF > 5%), 3.1M (12%) were low frequency (MAF between 1 and 5%) and 16.6M (62%) were rare (MAF < 1%) with a similar partition in all cohorts. Singletons variants (AC = 1) were >6M (24%) (Table 1 and Fig. 1b). For each individual, we identified on average ~3.5M variant sites including ~0.56M indels and ~7000 singletons. In comparisons with outbred references (EUR subset from 1000G Phase 3, the whole 1000G Phase 3 and UK10K) 34–45% of the INGI variants were not represented in samples of Northern European origin or in public sequence repositories (~12M with EUR, ~10M with 1000G and ~9M with UK10K, respectively): 89% of those variants are private to each INGI cohort. Moreover 8% of the sites shared between two or all three INGI cohorts were not found either in the whole 1000G or the EUR subpopulation from 1000G (which includes Italian samples from the Tuscany region - TSI), suggesting that they may be characteristic of the general Italian population but not captured by the only available Italian reference. The majority of the private variants are within the range of the low and rare frequencies (MAF < 1%) (Fig. 1c) while the proportion of low frequency and common variants are similar in the pool of shared sites (Supplementary Fig. 1, Supplementary Table 1).

**Table 1:** Final data release of WGS data for all the INGI cohorts

INGI All samples				
	CAR	FVG	VBI	INGI
Samples	124	378	424	926
Females	66	220	249	535
Males	58	158	175	391
Average coverage	6.31	7.23	6.12	6.55
Sites	13,370,262	17,002,010	19,361,094	26,619,091
Multiallelic sites	248,638	356,599	393,328	560,918
SNPs	12,208,629	15,521,313	17,830,208	24,557,366
INDELs	1,161,633	1,480,697	1,530,886	2,061,725
Sites MAF ≤ 1%	3,627,622	7,283,720	9,416,028	16,685,951
Sites 1% < MAF ≤ 5%	3,007,162	3,069,534	3,121,545	3,125,971
Sites MAF > 5%	6,735,478	6,648,756	6,823,521	7,123,064
Singletons SNPs	2,061,824	2,784,746	3,554,744	6,193,486
Singletons INDELs	92,372	131,275	133,156	273,679
Average heterozygosity rate per sample	17.57%	13.27%	12.16%	13.34%
Average derived allele count per sample	4,703,290	4,741,910	4,844,980	4,763,393
Average variations per sample	3,518,020	3,421,910	3,541,760	3,493,897
Average INDELs per sample	531,151	586,740	590,109	569,333
Average singleton per sample	17,285	7,671	8,646	6,925

The table shows information about the final data release for each INGI cohort separately as well as information on the pooled dataset (INGI column); sequence data were aligned to the Human genome reference build 37 (GRCh37)

### IGRP1.0: reference panel and imputation

**Fig. 2:**
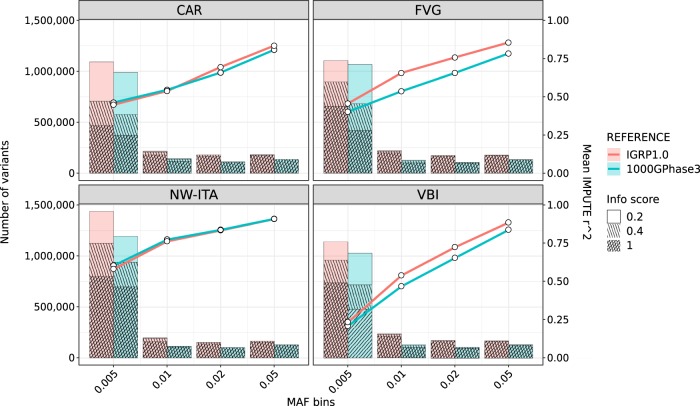
Imputation accuracy: mean values of r^2^ (right *y*-axes) stratified by minor allele frequency (coloured lines) and number of imputed sites (left *y*-axes) stratified by info score values and minor allele frequency (bar plot) for Italian cohorts. An outbred cohort from North Italy (NW-ITA) was included for comparison

After applying the filtering criteria explained in methods, we retained 95.6%, 94.29% and 92.06% of the variants for CAR, FVG and VBI, respectively (Supplementary Table 2). Merging our data with the 1000G Phase 3 haplotype reference panel yield 6.9M Italian population-specific variants or 7.8% of the IGRP1.0 panel (Supplementary Table 3). As shown in Fig. 2, the IGRP1.0 panel (red line) always outperforms the 1000G phase 3 reference panel for the INGI cohorts in terms of genotype concordance (*r*^2^ - right *y*-axes), while there is not a significant improvement for the outbred population (NW-ITA) (Supplementary Table 4). We compared our resource performances also in terms of the IMPUTE ‘info score’ metric. To discriminate between well and poorly imputed sites, in terms of their usefulness for GWAS analyses, we set a threshold of 0.4 for the info score metric, according to [13]. The proportion of well-imputed sites (info score ≥ 0.4) in the IGRP1.0 reference panel was higher compared with the 1000G Phase 3 reference panel (red and blue bars, respectively) at all frequencies tested, with an increase from 20 to 36% of rare sites (MAF ≤ 0.5%) with info score ≥ 0.4 (Fig. 2, Supplementary Table 5). The comparison of the two reference panels using an outbred Italian population shows a higher accuracy of IGRP1.0, respect to 1000G Phase 3. In particular, for the lowest frequency bin, we could impute 800,721 sites with IGRP1.0 versus 698,140 sites with 1000G phase 3 panel with info scores ≥ 0.4 and a 13% increase of reliably imputed rare sites. We further validated our resource on three Croatian cohorts (VIS, KORCULA, SPLIT): the IGRP1.0 panel has a higher proportion of well-imputed sites compared with other panels (Supplementary Fig. 2, Supplementary Tables 5 and 6). A direct comparison with the recent HRC reference panel [36] was not performed since a subset of the data presented in this paper (225 samples from the VBI cohort and 250 samples from the FVG cohort) is included in that reference. However, we checked the quality of sites belonging to the INGI cohorts but excluded because of filtering from the HRC reference: among seven test cohorts, we identified 696,895–624,434 polymorphic sites with an average proportion of good quality sites of 71% (63–81.5%). Focusing on rare variants for this subset, we can identify 256,222–326,076 polymorphic sites with a proportion of good quality sites between 15 and 63% (Supplementary Table 7). This last comparison demonstrates the excellent quality of the information added by our resource.

### IGRP1.0: GWAS studies

**Fig. 3:**
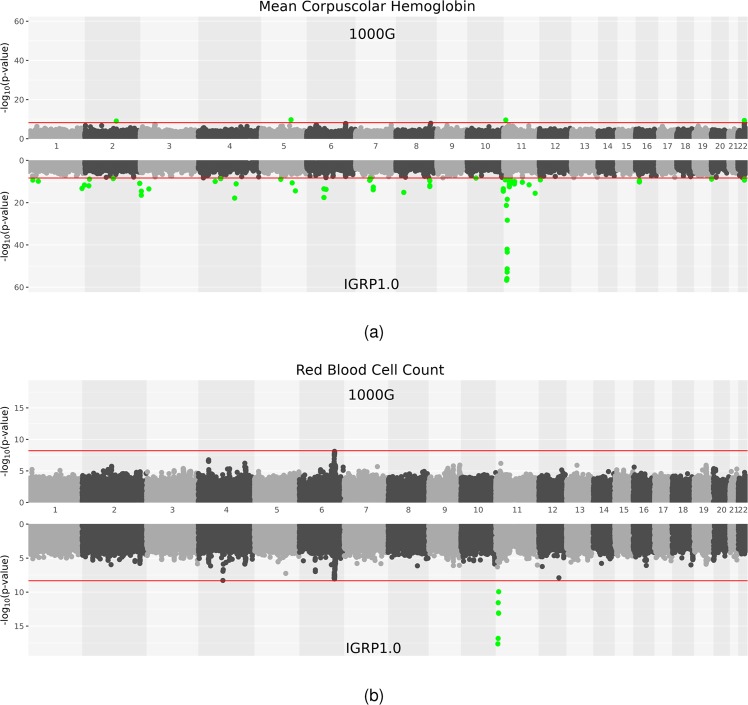
GWAS analyses: **a** Manhattan plot of GWAS meta-analysis on Mean Corpuscular Haemoglobin (MCH) phenotype: results in the bottom panel are from IGRP1.0 imputed data while on the top panel we show GWAS results obtained using the 1000G reference panel for imputation. **b** Manhattan plot of GWAS meta-analysis on Red Blood Cell Count (RBC) phenotype: results in the bottom panel are from IGRP1.0 imputed data while on the top panel we show GWAS results obtained using the 1000G reference panel for imputation

GWAS analyses using the different imputation panels comprised a total of 3292 individuals (Supplementary Table 8). Manhattan plots of all the meta-analysis results are shown in Supplementary Fig. 3. Lambda values of GWAS analyses showed no evidence for stratification (Supplementary Figs. 4 and 5). A meta-analysis of GWAS with 1000G showed significant results (*P* < 6.23 × 10^−9^) only for MCV and MCH (Supplementary Table 9). Overall, IGRP1.0 imputation panel allowed us to replicate known loci and loci identified through the 1000G imputation, also increasing the number of significant variants (i.e. in the *HBB* gene), as shown in Fig. 3a, b. Further details are reported in the Supplementary Notes, and the full results are reported in Supplementary Table 10. A direct comparison between the meta-analysis results (with *P* < 1 × 10^−5^), using the two different imputation panels and on the same markers, is reported in Supplementary Table 11.

### Population structure

**Fig. 4:**
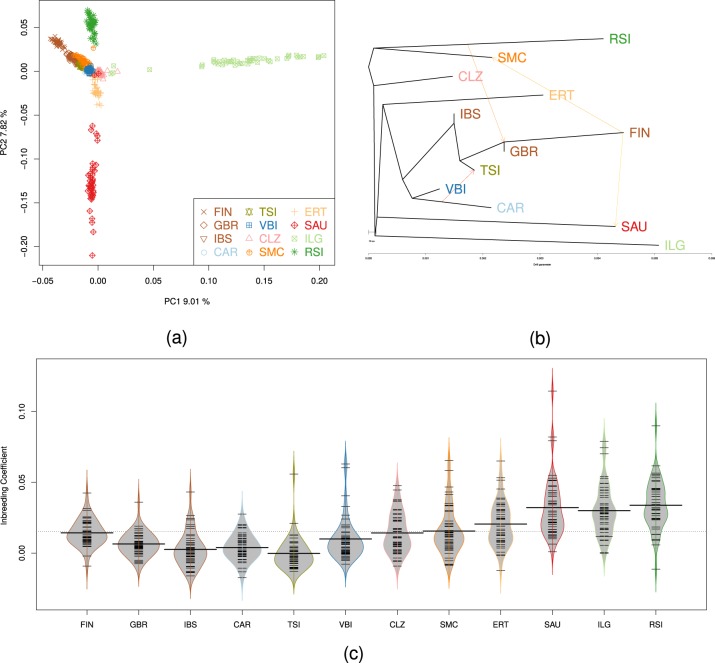
Population genetic analyses: **a** PCA of Italian samples and European 1000G populations using a subset of 46 individuals from each population. Variance explained by each axis is reported. Each population from FVG cohort - Erto (ERT), Illegio (ILG), Resia (RSI), Sauris (SAU), San Martino del Carso (SMC) and Clauzetto (CLZ) - are shown. The first axis separates ILG from all other Italian populations; the second axis separates SAU from RSI; Val Borbera (VBI) and Carlantino (CAR) cluster with Toscani in Italia (TSI), Finnish in Finland (FIN), British in England and Scotland (GBR), Iberian Population in Spain (IBS). **b** Treemix graph analyses with 3 migration edges: a link between North European populations and isolates such as RSI and SAU is shown; **c** Bean plots of Inbreeding coefficient of 1000G European populations and Italian populations. All FVG population have a higher inbreeding coefficient respect to other Italian and European population except for FIN. The plot shows that in the INGI populations the distribution of the inbreeding coefficient values are more sparse with respect to the actual reference Italian population of TSI from 1000G; each horizontal black bar represents an observation from the dataset

A PCA with seven European-ancestry populations showed how each INGI population separates from each other (in the first four principal components) (Fig. 4a and Supplementary Fig. 6). The separation of the six villages making up the FVG cohort - Erto (ERT), Illegio (ILG), Resia (RSI), Sauris (SAU), San Martino del Carso (SMC) and Clauzetto (CLZ) - demonstrates population structure and a high degree of isolation [12]. Analyses using pairwise genomic Fst (Supplementary Fig. 7) demonstrated a high level of differentiation between the six FVG villages. A further analysis was performed using Treemix [28] (Fig. 4b): this analysis showed evidence of gene flow between North European population and North Eastern Italians (showed by migration arrows in the graph). Admixture [37] analysis for *K* = 9 (solution with the lowest CV error) (Supplementary Fig. 8) highlighted that the more isolated FVG populations have ancestry components present at a low level in all European and Italian populations. Finally, the inbreeding coefficients and total homozygosity (due to ROH) showed high levels of variance among different Italian subpopulations as shown by the shape of the bean plots (Fig. 4c). The total homozygosity due to ROH and the total number of ROH segments discovered follow the same pattern (Supplementary Figs. 9 and 10) which is quite different from the TSI (Mann–Whitney *P* < 0.01).

### Natural selection

To identify markers and genes under selection, we first selected markers with |iHS| ≥ 2 as candidates [38]. Evidence of selection in all Italian cohorts was found for 37 genes. However, as shown in Supplementary Fig. 11, the major part of genes in INGI cohorts with signatures of selection did not harbour signals in the TSI. Specifically, the fraction of genes under selection only in one INGI population ranges from 74% in VBI to 86% in RSI, respect to TSI. Is interesting to note that *FHIT*, *CSMD1*, *CNTNAP2*, *MACROD2*, *RBFOX1* and *PTPRD* shared selection signature among all cohorts but with signals on different markers. Besides, we used a more stringent cut-off for selection using as criteria a |iHS| ≥ 2.5, discovering a total of 397 genes with signatures of selection. Among them only 15 harbour signatures of selection in all Italian cohorts. Using more stringent criteria follows the pattern observed using the less stringent one (20 SNPs with at least iHS > = 2). A complete list of the genes found with different cut-off is reported in Supplementary Tables 12 and 13, and additional details are reported in Supplementary Notes

### Deleterious variants enrichment

In order to display the different deleterious or neutral variant distribution compared with the Italian reference population, we applied the DVxy statistic [12] for DV variants (Drifted Variants respect to a reference) between 1–2 AC and 3–5 AC in each population, using TSI as the Italian reference population then we grouped variants according to CADD score. In our analysis, we discovered a significant relative enrichment in deleterious variants with CADD ≥ 20 in the more isolated populations (ILG, RSI, SAU and also SMC) compared with the TSI (DVxy-sd > 1). However, we found no differences when considering variants with low CADD (CADD 0-5, DVxy +/− sd = 1) for variants between 3–5 AC (Supplementary Fig. 12). A similar pattern was found for DV variants between 1–2 AC (Supplementary Fig. 13). In order to describe the distribution of DV variants (between 3–5 AC and CADD ≥ 20), we estimated the ratio between variants that are drifted in only one population but not in another and variants that are drifted in both (DV ratio) (Supplementary Fig. 14), this was done for all possible pairs. All values are highly positive, indicating that the majority of drifted deleterious variants are population-specific.

### Human knockout

In our cohorts, we found 506 LoF presenting with a CADD ≥ 20 at homozygous state in at least one individual per population (Supplementary Table 14). Gene ontology analysis revealed an excess of transmembrane signalling receptor genes, including olfactory receptors, as already described [39]. The number of variants considered TOTAL putative LoF is 205, overlapping 195 different genes. Additional details on the HKOs found in the INGI cohorts are reported in the Supplementary Notes and Supplementary Table 15. Among the whole LoF set (TOTAL and PARTIAL), we analysed only variants reported in gnomAD (to avoid the chance of false positives), overlapping 133 different genes, which are distributed among the cohorts as shown in Supplementary Fig. 15. We found that the majority of genes in which HKO were detected are unique of FVG, VBI and CAR (61, 36 and 10, respectively) whereas only 13 genes are shared among all populations. Among these HKO genes, only two show evidence of selection in the same population in which the HKO carriers are present (see Supplementary Table 16). The majority of the RVIS score (residual variation intolerance score) [35] for the whole set of HKO genes are positive (median = 0.73) and significantly different (Wilcoxon–Mann–Whitney *P* = 7 × 10^−55^) from the whole set of genes reported (median = −0.05).

## Discussion

The ability to interrogate all classes of genetic variations is critical for the classification of genetic determinants of complex and monogenic disorders: the whole-genome sequencing of populations such as isolates has given a significant contribution [40]. Here, we report the results of the analyses obtained through the investigation of WGS from 947 subjects coming from different Italian geographic areas (i.e. South, North-West and North-East) and their contribution to the identification and description of a significant proportion of the Italian population pool of genetic variation. The number of new variants described, confirms that these genomes can increment the catalogue of Italian genotypic variation, in particular in the low-frequency spectrum. The INGI custom reference panel (IGRP1.0) outperformed the 1000G Phase 3 reference for imputation of inbred and outbred Italian and other European populations such as the Croatians cohorts. At the time of writing, the “gold standard” for imputation reference panel is represented by the HRC dataset, but we could not carry out a direct comparison with the HRC panel since a subset of the samples we present here are included in that resource. We were able to assess the excellent quality of the information added by our complete data by taking into account only those variants belonging to the INGI cohorts and not represented in the HRC panel. As already shown in previous works [13–15], the addition of study-specific WGS data increases accuracy of imputation for low-frequency variants (MAF < 1%), providing a cost-effective way to improve power and resolution for GWAS studies and help the identification of population-specific variants of different Italian and possibly Southern European populations: notably, we are incrementing the total number of variants that are valuable for GWAS studies in INGI populations as expected, and furthermore, in other outbred populations in terms of imputation quality, confirming, as already shown in [1, 16] the advantages of ethnically matched reference panels. With this resource at our disposal, another question arises: will we be able to increment the power to detect genome-wide significant loci/variants using this new reference panel for imputation? In this case, the reliability of IGRP1.0 panel was proven running a series of GWAS tests on some selected RBC traits. GWAS studies carried out with IGRP1.0 panel imputed data, not only replicated previous findings yielding high statistical significance, but they also demonstrated that several previously found suggestive signals (*P* < 1 × 10^−5^) became genome-wide significant (*P* < 1 × 10^−8^). Furthermore, we discovered additional signals arising from variants not present in the previous imputation (i.e. the beta thalassaemia-related variant GRCh37 chr11:g.5248004G > A - rs11549407). and two variants significantly associated to MCV and MCH traits in *RBFOX1*, that we were able to pinpoint only through our custom reference panel. Nonetheless, caution and further studies will be needed to assess the role of the suggestive signals. Recently published results based on array data pinpointed the genetic diversity in the Italian peninsula [6] along with the presence of isolates [8]. These insights showed the lack of homogeneity of genomes coming from different regions of Italy in terms of diverse genomic aspects (population structure, natural selection signatures, deleterious variants distribution and HKO) and, as a consequence, how the usage of only one reference population for Italians, such as the Tuscans (TSI), is not reliable. We confirmed the non-homogeneous genetic background of the Italian populations from North to South. Our analyses using WGS not only recapitulate what was previously mentioned [8, 11] but add a new degree of detail due to the number of markers used. This degree of detail is particularly appreciated, for example, in the total number of ROH discovered (which could highlight different regions covered by ROH in different populations). Previous works on Italian samples showed the presence of different isolate through the territory [8–10]. We can suppose that the presence of small villages with different level of isolation could be more common than expected in Italy and for this reason, understanding the various characteristics of each isolate is essential to provide a better picture of the genomic variation in the Italian peninsula. An exploration of natural selection demonstrated that environmental differences along the peninsula might have shaped the genome through mechanisms such as evolution and selective pressure [6]. Our analyses pinpointed the presence of shared selective pressure in genes in all Italian populations but also on the level of selection signatures that are private to single populations (when substructure is taken into account) ranging up to 86% of the total genes found for RSI (with iHs cut-off of 2). Considering the relationship of some populations (RSI, SAU, SMC) with North European populations (as shown in Treemix analyses), we can hypothesise that a number of haplotypes passed in some North-East Italian populations but not in others: this peculiar gene flow could be responsible for some unique signals of selection. However, the presence of different selection signals could also not be caused by environmental differences, but they are due to shared selection pressure with the ancestral population and are retained only in some villages after the founder effect. For what concerns the distribution of deleterious variants, the relative relaxation of purifying selection in the presence of isolation, leading to an increased frequency of specific deleterious variants has already been demonstrated [12, 41]. This aspect reinforces our thesis about the need of a more broadened reference for the Italian genomic variation, as we demonstrated that not only do we have an enrichment of low-frequency deleterious variants (CADD ≥ 20) in our genomes, but also most of this enrichment is population-specific. In our analyses of HKOs, we discovered that the majority of genes harbouring HKO are private of each cohort, and many of them (91%) were not found in any selection scan, suggesting the lack of evolutionary constraints for these genes. The average positive RVIS score distribution for these genes further confirms this hypothesis. This result gives us another hint of the necessity of multiple genomes to describe the catalogue of HKO present in Italy. Furthermore, HKO and pattern of deleterious variants are useful examples to show how clinical-relevant polymorphisms could be found enriched in frequency in specific populations within the same country. In conclusion, we showed how our unique dataset of populations and WGS data enhance the content of publicly available human WG data sets (i.e. 1000G, gnomAD databases), in which Southern European populations - a significant proportion of the overall European populations - are highly underrepresented, and that this resource will enable to produce regionally appropriate reference panels. Furthermore, since in Italy the effort to build a National Genomic BioBank is not in place yet, the availability of a catalogue of rare and low-frequency variants for Italian populations will facilitate the understanding of these genetic loci, improving the accuracy and efficacy of a series of genetics/genomics studies, and subsequently opening new perspectives for precise medicine and drug targets identification.

## Supplementary information

Supplementary Figure Legends

Supplementary Notes

Supplementary Table 1

Supplementary Table 2

Supplementary Table 3

Supplementary Table 4

Supplementary Table 5

Supplementary Table 6

Supplementary Table 7

Supplementary Table 8

Supplementary Table 9

Supplementary Table 10

Supplementary Table 11

Supplementary Table 12

Supplementary Table 13

Supplementary Table 14

Supplementary Table 15

Supplementary Table 16

Supplementary Table 17

Supplementary Table 18

Supplementary Figure 1

Supplementary Figure 2

Supplementary Figure 3

Supplementary Figure 4

Supplementary Figure 5

Supplementary Figure 6

Supplementary Figure 7

Supplementary Figure 8

Supplementary Figure 9

Supplementary Figure 10

Supplementary Figure 11

Supplementary Figure 12

Supplementary Figure 13

Supplementary Figure 14

Supplementary Figure 15

## References

[1] Gudbjartsson DF, Helgason H, Gudjonsson SA, Zink F, Oddson A, Gylfason A (2015). Large-scale whole-genome sequencing of the Icelandic population. Nat Genet..

[2] The 1000 Genomes Project Consortium. (2015). A global reference for human genetic variation. Nature..

[3] The UK10K Consortium. (2015). The UK10K project identifies rare variants in health and disease. Nature..

[4] Karczewski KJ, Weisburd B, Thomas B, Solomonson M, Ruderfer DM, Kavanagh D (2017). The ExAC browser: displaying reference data information from over 60 000 exomes. Nucleic Acids Res.

[5] The ENCODE Project Consortium. (2007). Identification and analysis of functional elements in 1% of the human genome by the ENCODE pilot project. Nature..

[6] Sazzini M, Gnecchi Ruscone GA, Giuliani C, Sarno S, Quagliariello A, De Fanti S (2016). Complex interplay between neutral and adaptive evolution shaped differential genomic background and disease susceptibility along the Italian peninsula. Sci Rep..

[7] Bonifazi C, Heins F (2000). Long-term trends of internal migration in Italy. Int J Popul Geogr.

[8] Esko T, Mezzavilla M, Nelis M, Borel C, Debniak T, Jakkula E (2013). Genetic characterization of northeastern Italian population isolates in the context of broader European genetic diversity. Eur J Hum Genet.

[9] Izzi C, Sanna-Cherchi S, Prati E, Belleri R, Remedio A, Tardanico R (2006). Familial aggregation of primary glomerulonephritis in an Italian population isolate: Valtrompia study. Kidney Int.

[10] Messina F, Scorrano G, Labarga CM, Rolfo MF, Rickards O (2010). Mitochondrial DNA variation in an isolated area of Central Italy. Ann Hum Biol.

[11] Colonna V, Pistis G, Bomba L, Mona S, Matullo G, Boano R (2013). Small effective population size and genetic homogeneity in the Val Borbera isolate. Eur J Hum Genet.

[12] Xue Y, Mezzavilla M, Haber M, McCarthy S, Chen Y, Narasimhan V, et al. Enrichment of low-frequency functional variants revealed by whole-genome sequencing of multiple isolated European populations. Nat Commun. 2017. https://www.ncbi.nlm.nih.gov/pmc/articles/PMC5490002/. 10.1038/ncomms15927 PMC5490002 28643794

[13] Pistis G, Porcu E, Vrieze SI, Sidore C, Steri M, Danjou F (2015). Rare variant genotype imputation with thousands of study-specific whole-genome sequences: implications for cost-effective study designs. Eur J Hum Genet.

[14] Mitt M, Kals M, Pärn K, Gabriel SB, Lander ES, Palotie A (2017). Improved imputation accuracy of rare and low-frequency variants using population-specific high-coverage WGS-based imputation reference panel. Eur J Hum Genet.

[15] Deelen P, Menelaou A, van Leeuwen EM, Kanterakis A, van Dijk F, Medina-Gomez C (2014). Improved imputation quality of low-frequency and rare variants in European samples using the ‘Genome of The Netherlands. Eur J Hum Genet.

[16] Sidore C, Busonero F, Maschio A, Porcu E, Naitza S, Zoledziewska M, et al. Genome sequencing elucidates Sardinian genetic architecture and augments association analyses for lipid and blood inflammatory markers. Nat Genet. 2015. http://www.nature.com/ng/journal/vaop/ncurrent/full/ng.3368.html. 10.1038/ng.3368 PMC4627508 26366554

[17] Li H, Durbin R (2010). Fast and accurate long-read alignment with Burrows–Wheeler transform. Bioinformatics..

[18] McLaren W, Gil L, Hunt SE, Riat HS, Ritchie GRS, Thormann A (2016). The ensembl variant effect predictor. Genome Biol..

[19] Marchini J, Howie B (2010). Genotype imputation for genome-wide association studies. Nat Rev Genet.

[20] Aulchenko YS, Ripke S, Isaacs A, van Duijn CM (2007). GenABEL: an R library for genome-wide association analysis. Bioinforma Oxf Engl.

[21] Willer CJ, Li Y, Abecasis GR (2010). METAL: fast and efficient meta-analysis of genomewide association scans. Bioinforma Oxf Engl.

[22] Del-Aguila JL, Beitelshees AL, Cooper-DeHoff RM, Chapman AB, Gums JG, Bailey K (2014). Genome-wide association analyses suggest NELL1 influences adverse metabolic response to HCTZ in African Americans. Pharmacogenomics J.

[23] Kerns SL, Dorling L, Fachal L, Bentzen S, Pharoah PDP, Barnes DR (2016). Meta-analysis of genome wide association studies identifies genetic markers of late toxicity following radiotherapy for prostate cancer. EBioMedicine..

[24] Turner S. qqman: Q-Q and Manhattan plots for GWAS data. 2017. https://CRAN.R-project.org/package=qqman.

[25] Lucas A. An R package for creating mirrored Manhattan plots: anastasia-lucas/hudson. 2018. https://github.com/anastasia-lucas/hudson.

[26] Daly M, Purcell S, Neale B, Toddbrown K, Thomas L, Ferreira M (2007). PLINK: a tool set for whole-genome association and population-based linkage analyses. Am J Hum Genet.

[27] Benazzo A, Panziera A, Bertorelle G (2015). 4P: fast computing of population genetics statistics from large DNA polymorphism panels. Ecol Evol..

[28] Pickrell JK, Pritchard JK (2012). Inference of population splits and mixtures from genome-wide allele frequency data. PLOS Genet..

[29] Kullo IJ, Ding K, Jouni H, Smith CY, Chute CG (2010). A genome-wide association study of red blood cell traits using the electronic medical record. PLOS One..

[30] Voight BF, Kudaravalli S, Wen X, Pritchard JK (2006). A map of recent positive selection in the human genome. PLOS Biol.

[31] Szpiech ZA, Hernandez RD (2014). selscan: an efficient multithreaded program to perform EHH-based scans for positive selection. Mol Biol Evol.

[32] Kircher M, Witten DM, Jain P, O’Roak BJ, Cooper GM, Shendure J (2014). A general framework for estimating the relative pathogenicity of human genetic variants. Nat Genet.

[33] Narasimhan VM, Hunt KA, Mason D, Baker CL, Karczewski KJ, Barnes MR (2016). Health and population effects of rare gene knockouts in adult humans with related parents. Science..

[34] Chen H. VennDiagram: generate high-resolution Venn and Euler Plots. 2018. https://CRAN.R-project.org/package=VennDiagram.

[35] Petrovski S, Wang Q, Heinzen EL, Allen AS, Goldstein DB (2013). Genic intolerance to functional variation and the interpretation of personal genomes. PLOS Genet..

[36] McCarthy S, Das S, Kretzschmar W, Delaneau O, Wood AR, the Haplotype Reference Consortium (2016). A reference panel of 64,976 haplotypes for genotype imputation. Nat Genet.

[37] Alexander DH, Novembre J, Lange K (2009). Fast model-based estimation of ancestry in unrelated individuals. Genome Res.

[38] Pickrell JK, Coop G, Novembre J, Kudaravalli S, Li JZ, Absher D (2009). Signals of recent positive selection in a worldwide sample of human populations. Genome Res..

[39] MacArthur DG, Balasubramanian S, Frankish A, Huang N, Morris J, Walter K (2012). A systematic survey of loss-of-function variants in human protein-coding genes. Science..

[40] Hatzikotoulas K, Gilly A, Zeggini E (2014). Using population isolates in genetic association studies. Brief Funct Genom.

[41] Chheda H, Palta P, Pirinen M, McCarthy S, Walter K, Koskinen S, et al. Whole-genome view of the consequences of a population bottleneck using 2926 genome sequences from Finland and United Kingdom. Eur J Hum Genet. 2017. http://www.nature.com/ejhg/journal/vaop/ncurrent/full/ejhg2016205a.html. 10.1038/ejhg.2016.205 PMC5346294 28145424

