# Supplementary material for: A bird’s-eye view of Italian genomic variation through whole-genome sequencing
Source: Eur J Hum Genet. 2019 Nov 29;28(4):435–44. doi: 10.1038/s41431-019-0551-x (PMC7080768; doi:10.1038/s41431-019-0551-x)
Supplement: Supplementary file 1 — Supplementary Figure Legends [file 41431_2019_551_MOESM1_ESM.docx]

**Supplemental Legends**

**Supplementary Figure 1.** The stacked bar-plot represent the number of shared variants between the whole INGI dataset and outbred populations, stratified by Minor Allele Frequency. Singletons sites (AC=1) are included.

**Supplementary Figure 2.** Imputation accuracy: mean values of r^2^ (right y-axes) stratified by minor allele frequency (coloured lines) and the number of imputed sites (left y-axes) stratified by info score values and minor allele frequency (bar plot) for Croatian cohorts.

**Supplementary Figure 3.** GWAS analyses: Manhattan plot of meta-analyses comparison for all analysed red blood cell traits using a) 1000G reference panel and b) IGRP1.0.

**Supplementary Figure 4.** GWAS analyses: Lambda values of GWAS analyses for all analysed red blood cell traits using 1000G reference panel.

**Supplementary Figure 5.** GWAS analyses: Lambda values of GWAS analyses for all analysed red blood cell traits using IGRP1.0 reference panel.

**Supplementary Figure 6.** PCA of Italian samples and European 1000G populations using a subset of 46 individuals from each population (3rd PC versus 4th PC ). Variance explained by the axis is reported. Each population from FVG cohort - Erto (ERT), Illegio (ILG), Resia (RSI), Sauris (SAU), San Martino del Carso (SMC) and Clauzetto (CLZ) - has its own axis of variation; Val Borbera (VBI) and Carlantino (CAR) cluster with Toscani in Italia (TSI), Finnish in Finland (FIN), British in England and Scotland (GBR), Iberian Population in Spain (IBS).

**Supplementary Figure 7.** Pairwise matrix of Fst of Italian samples (Erto - ERT, Illegio - ILG, Resia - RSI, Sauris - SAU, San Martino del Carso - SMC, Clauzetto - CLZ, Val Borbera - VBI, Carlantino - CAR) and 1000G populations; Super-population groups from 1000G are splitted in sub-populations to add detail on Fst distribution: ITU (Indian), GWJ (Gambian Jola), CHS (Southern Han Chinese), GBR (British), KHV (Kinh Vietnamese), STU (Sri Lankan), CHD (Denver Chinese), PUR (Puerto Rican), FIN (Finnish), ACB (African-Caribbean), GWD (Gambian Mandinka), MSL (Mende), ESN (Esan), MXL (Mexican-American), CLM (Colombian), PEL (Peruvian), TSI (Tuscan), PJL (Punjabi), IBS (Spanish), BEB (Bengali), ASW (African-American SW), GWW (Gambian Wolof), YRI (Yoruba), LWK (Luhya), CEU (CEPH), JPT (Japanese), CDX (Dai Chinese), CHB (Han Chinese), GIH (Gujarati), GWF (Gambian Fula).

**Supplementary Figure 8.** Admixture analysis: Ancestry proportions of the studied individuals as revealed by ADMIXTURE with K=9. A stacked column of the K proportions represents each individual. Val Borbera (VBI) shows an admixture pattern similar to that of 1000 Genome Tuscan (TSI), CAR shows a violet component that is shared with other Italian populations and with IBS, while FVG populations show their own ancestral component (except for CLZ). Interestingly SMC shows an orange component shared with FIN and GBR which is present at different fractions in all European and Italian populations.

**Supplementary Figure 9.** ROH analysis: Distribution of the total amount of homozygosity in all cohorts. Minimum ROH length was set at 1 Mb.

**Supplementary Figure 10.** ROH analysis: Distribution of the number of homozygosity segments in all cohorts. Minimum ROH length was set at 1 Mb.

**Supplementary Figure 11.** Natural selection: Proportion of genes with signatures of selection in INGI subpopulations respect to TSI. Blue colour represents the fraction of shared genes with TSI, orange colour represents the fraction of genes found under selection only in the isolate and not in TSI.

**Supplementary Figure 12.** Deleterious variant enrichment: DVxy statistic for each INGI cohort using as reference the TSI population using variants between 3-5 allele count (AC) and binned for CADD score as shown in the x-axis. Confidence intervals were created using the distribution of all 22 chromosomes and represent one standard deviation. A value equal to 1 means no enrichment, a value minor than 1 means depletion in the INGI cohort respect to TSI and a value greater than 1 means enrichment with respect to the reference.

**Supplementary Figure 13.** Deleterious variant enrichment: DVxy statistic for each INGI cohort using as reference the TSI population using variants between 1-2 allele count (AC) and binned for CADD score as shown in the x-axis. Confidence intervals were created using the distribution of all 22 chromosomes and represent one standard deviation. A value equal to 1 means no enrichment, a value minor than 1 means depletion in the INGI cohort respect to TSI and a value greater than 1 means enrichment with respect to the reference.

**Supplementary Figure 14.** Heatmap of the ratio of DV variants defined as the ratio of DV variants that are different between pairs of INGI sub-populations and DV variants that are shared between INGI pairs. DV variants are defined as follows: variants with 3-5 AC, CADD ≥ 20, frequency fold enrichment ≥ 3 with respect to the Italian reference (TSI).

**Supplementary Figure 15.** HKOs distribution among cohorts: Venn diagram shows how the 133 genes harbouring HKO variants (with at least 1 homozygous individual) are distributed among INGI cohorts.
