# Supplementary material for: A bird’s-eye view of Italian genomic variation through whole-genome sequencing
Source: Eur J Hum Genet. 2019 Nov 29;28(4):435–44. doi: 10.1038/s41431-019-0551-x (PMC7080768; doi:10.1038/s41431-019-0551-x)
Supplement: Supplementary file 2 — Supplementary Notes [file 41431_2019_551_MOESM2_ESM.docx]

**Supplementary Notes: A** **bird's-eye view of Italian genomic variation through Whole Genome Sequencing**

1. **A short overview of the history for the villages from the Friuli-Venezia Giulia, Val Borbera and Carlantino**
2. **WGS data generation: variant calling and quality control**
3. **Reference imputation panel**
4. **Reference panel quality assessment**
5. **Genome-wide association studies (GWAS)**
6. **Population structure**
7. **The Pattern of Natural Selection in Italian Populations**
8. **Human Knock Out**
9. **Data availability**

# **1. A Short overview of the history for the villages from the Friuli-Venezia Giulia, Val Borbera and Carlantino**

# **CLAUZETTO (CLZ)** **-** GPS coordinates: 46°14′N 12°55′E

The village of Clauzetto is located at 570 meters above the sea level. The spoken dialect is Friulano, which is the standard dialect for FVG region. The latest census revealed a population of 377 people. The earliest mentioning of Clauzetto dates to the XII century AD.

**ERTO (ERT) -** GPS coordinates: 46°17′N 12°22′E

The village of Erto was built in the valley of Vajont (750 meters above sea level). The population size of Erto is around 370 inhabitants.

The spoken language is a local dialect, which has colloquially named as Ertano. In the year of 1963, the local dam of Vajont broke, and the flood killed many inhabitants of Erto. According to a local legend, the village was founded by the survivors of a Cimbri colony around the first century BC.

**ILLEGIO (ILG) -** GPS coordinates:46°24′N 13°01′E

Illegio is a relatively small village. It is located in an isolated valley (529 meters above sea level). Due to its isolated position, the consanguineous marriages have been usual. There are a minimal number of surnames; this is indicative for almost no immigration. According to the latest census the population size was 424 inhabitants.

Near the village of Illegio is the parish church of St. Florian, which dates back to late IV century AD that could give hints of the foundation of this village.

**SAN MARTINO del CARSO (SMC) -** GPS coordinates: 45°52′38″N 13°32′10″E

The village of San Martino del Carso (SMC) is located on the base of the mountain San Michele (1444 meters above sea level). During World War II the village suffered considerable damage, and according to the latest census the population size of SMC was 258 inhabitants.

**RESIA(RSI) -** GPS coordinates:46°23′N 13°18′E

The village of Resia is located in the valley of Resia which has been historically very isolated. The inhabitants of the village of Resia are speaking an archaic form of Slovenian language, known as Resian dialect. Resians (as they are called the inhabitants) are culturally very close to Slovenians. It seems the village was founded around the VII century from unknown populations. The village was nearly destroyed during the earthquake of 1976. The level of consanguineous marriages has been very high.

**SAURIS (SAU) -** GPS coordinates:46°27′58.46″N 12°42′30.67″E

The village of Saudis is located in the geographically isolated valley of Lumiei (977 meters above sea level). The village was built around the XIII century as the first historical documents date back to this time. The inhabitants are speaking the language Saurana, Saurana is an ancient German dialect. According to the latest census the population size of Sauris was 429 inhabitants.

**CARLANTINO (CAR) -** GPS coordinates:41°35′40.51″N 14°58′31.49″E

The village is located at 558 meters above sea level and was built on the of a hill over the lake of Occhito. The foundation of this village began around 1558 when a noble who received the task to build a settlement in the Land of Celenza. It underwent substantial emigration during the 1970s, and many inhabitants moved to South America. The latest census revealed a population of 941 individuals.

**VAL BORBERA (VBI) -** GPS coordinates:44°37′15″N 9°10′33″E

The Val Borbera is a broad valley with surrounding mountains. There are a total of seven villages located around 400 to 800 meters on the sea level.

It was subject to substantial emigration during 1970, and the actual census size of the valley is around ~3000. The inhabitants are called ValBorberini.

The Borbera valley was initially inhabited since the Iron Age (~ 1000 BC). Around the ~30 BC, the valley was occupied by the Romans. In the subsequent ages the valley was invaded and it was the first part of the dominions of Odoacre (476-493) then was invaded by the Visigoths around 493 and then by the Lombards around 570. During 1740 was annexed to the Sardinia Kingdom and them in 1861 became part of Italy.

**2.WGS data generation: variant calling and quality control**

All samples selected for sequencing had already genotype data from other platforms (SNP arrays and exome chip genotyping arrays for all three cohorts): these data allowed us to assess genotype concordance against a “trusted” set of variants. We selected the samples randomly for all cohorts. **Supplementary Table 17** summarises the total number of samples sequenced in each of the three sequencing centres: the Wellcome Trust Sanger Institute in Hinxton (UK), BGI, Shenzhen (PRC) and the San Raffaele Hospital (HSR) in Milan. All the data were post-processed at the Sanger Institute. Written informed consent for participation was obtained from all subjects. Regarding the FVG cohort, the project was approved by the Ethical committee of the IRCCS Burlo-Garofolo. Regarding the CAR cohort, the project was approved by the local administration of Carlantino, the Health Service of Foggia Province, and the ethical committee of the IRCCS Burlo-Garofolo of Trieste. For the VBI cohort, data collection and genotyping were approved by the institutional ethical committee of the San Raffaele Hospital in Milan and by the Regione Piemonte.

The raw data were checked first at lane level to remove any sample with bad quality data. Fifty-four samples were realigned to the hs37d5 reference sequence since they were aligned to a previous version of the GRCh37 build; this process has been carried out using the ‘Bridgebuilder system’ developed by the Human Genetics Informatics group at the Wellcome Trust Sanger Institute [1].

After the alignment, performed with *bwa* software, Picard MarkDuplicates [2] is run to mark any Optical and PCR duplicates. Each aligned and duplicate-marked bam file was then improved through the implementation of the following steps: 1) Realignment around already knew and discovered INDELs using GATK [3] RealignerTargetCreator and IndelRealigner; 2) Base Quality Recalibration by GATK BQSR using the BaseRecalibrator and PrintReads tools; 3) Recalculation of the MD tag by *samtools* [4] calmd; 4) Bam indexing.

For the Carlantino cohort, sequencing was carried out using Illumina technology (Genome Analyzer and HiSeq 2000) at the Wellcome Trust Sanger Institute for 115 samples with an average coverage of 4x, an additional batch of 40 samples was sequenced at Beijing Genomics Institute (BGI) with an average coverage of 10x. Among the 115 samples sequenced at the Sanger Institute, 27 failed the quality check at the lane level: 5 were re-processed while 22 were excluded from further analyses.

The most common cause of failure was the high percentage of adapter contamination and a bimodal insert size distribution.

For the Friuli Venezia Giulia cohort, 200 samples were sequenced at the Wellcome Trust Sanger Institute with a mean coverage of 4x and 192 samples at BGI with a mean coverage of 10x. Among the 200 Sanger samples, only 4 failed the quality check at the lane level and excluded from further analyses. Among the BGI set, six samples were duplicated from the Sanger pool: we merged the two sets of data in order to increase the coverage of each sample. We removed one additional sample from this set due to data corruption.

The data for the Val Borbera Cohort were generated at a mean coverage of 6x for all selected samples: 210 were sequenced at the Wellcome Trust Sanger Institute, 209 were sequenced at BGI, and a small batch of 29 was processed at the San Raffaele Hospital. After the first step of the quality check we removed two samples from the Sanger Institute set for contamination and low-quality DNA respectively, 12 samples from the OSR dataset for bad quality and 1 sample from the BGI set for data corruption.

Finally, a total of 947 samples was sent forward for the Variant Calling step.

We separately produced genotype calls for autosomal chromosomes for each population using the Human genome reference build 37 (GRCh37) with the pipeline described below.

Samtools mpileup (v.1.2) [4] was used for multisample genotype calling (parameter set: -E -t DP,DV,SP -C50 -pm3 -F0.2 -d 10000). The generated BCF files were converted to VCF format with bcftools call (v.1.2) (parameter set: -Nvm) and filtered with bcftools filter (v.1.2) (parameter set: -m+ -sLowQual -e"%QUAL<=0"-g3 -G10 -Ov - ). We applied the Variant Quality Score Recalibrator (VQSR) filtering to the raw call data with GATK v.3.3 [5]. Raw calls from samtools for SNVs and INDELs were used separately with the UnifiedGenotyper module in "Given allele mode" to generate all the annotation needed to calculate the VQSLOD scores through the VariantRecalibrator module. For SNVs we selected the following parameters: i) Annotations: QD, DP, FS, HaplotypeScore, MQRankSum, ReadPosRankSum, InbreedingCoeff; ii) Training set: HapMap 3.3, Omni 2.5M chip, 1000 Genomes Phase I; iii) Truth set: HapMap 3.3, Omni 2.5M chip; iv) Known set: dbSNP build 138. For INDELs we selected: i) Annotations: DP, FS, ReadPosRankSum, MQRankSum; ii) Training set: Mills-Devine, 1000 Genomes Phase I, dbSNP v138; iii) Truth set: Mills-Devine; iv) Known set: Mills-Devine, dbSNP build 138. For each population, we chose the lowest VQSLOD threshold according to the output produced by VariantRecalibrator to select the best cut-off in terms of specificity and sensitivity of the trained model. The Transition/Transversion (Ti/Tv) ratio was used as a parameter to select the best threshold, taking as reference the value of ∼ 2 calculated by [6]. For SNPs the minimum VQSLOD values selected were -59.1994 (99.94% truth sensitivity threshold), -15.0283 (99.80% truth sensitivity threshold), -22.6034 (99.9% truth sensitivity threshold) for VBI, FVG and CAR cohort respectively. For INDELs, we used a more conservative approach, selecting a sensitivity threshold of 95% for each population. The filter was applied to each call set with GATK ApplyRecalibration module.

We performed several genotype refinement steps on the filtered data: 1) BEAGLEv4.r1230 [7] was used to assign posterior probabilities to all remaining genotypes. 2) SHAPEITv2 [8] to phase all genotypes calls and 3) IMPUTEv2 [9] to perform internal imputation in order to correct genotyping errors.

Finally, bcftools annotate (v.1.2) was used to add information about Ancestral Allele and allele frequencies from 1000G phase 3 [10] populations and rsIDs from dbSNP v.141 [11]. The Variant Effect Predictor v.90 [12] provided all consequence annotation as well as Polyphen and Sift information. CADD score information was also added.

Samples and sites were again investigated for outliers or artefacts after the variant calling.

First, we looked for batch effects due to the different sequencing centres: we conducted an MDS analysis on each cohort testing the first PCA component for correlation with the sequencing centre variable with a Pearson’s correlation test and obtaining a significant outcome only for the FVG cohort (P=0.001728). We compared the analysis for the FVG cohort with data available from a previous work, showing that the pattern is consistent with the underlying population structure. We then generated a sites exclusion list. We focused on a) Hardy-Weinberg equilibrium (sites removed if exact test p-value ≤1x10^−8^); b) Heterozygosity rate distribution (removed sites with values greater than 3 standard deviations of the mean); c) MAF mismatch when compared with SNP array data; d) Non Reference Discordance rate (NRDR), defined as the ratio between the sum of concordant calls of the alternative allele in WGS and array data, and the sum of all discordant calls of the alternative allele in WGS and array data (cut off value for removal of 3 standard deviations of the mean).

We removed 5 552, 2 577 and 2 502 sites from CAR, FVG and VBI respectively. The sample quality assessment led to the removal of three samples from the FVG cohort, nine samples from the CAR cohort and nine samples from the VBI cohort.

We excluded samples using the following parameters: a) Singleton number, b) Heterozygosity rate and c) Non Reference Discordance rate. We removed one sample from the FVG cohort for an excess of singletons (~100 000 singletons counted). We also calculated the heterozygosity rate for each sample and removed all samples with values exceeding a threshold of 3 SD from the average value for each population: one sample was removed from the CAR cohort, one sample from the FVG cohort and four samples from the VBI cohort. Finally, we calculated the samples’ non-reference discordance rate and removed all individuals with an NRDR greater than 5%: 8 samples from the CAR cohort, one sample from FVG cohort and five samples from the VBI cohort. Average values for NRDR are reported in **Supplementary Table 18.**

**3. Reference imputation panel**

We selected a ‘highly reliable’ subset of variants to include in our reference panel.

In order to avoid mismatches between the INGI datasets, we split all multi-allelic variant sites in different vcf records and performed INDELs normalisation with the *bcftools* *norm* tool to prepare the data. We processed the data from 1000G Project phase 3, and the UK10K project in the same way.

The criteria described in Material and Methods were used to build a ‘core’ INGI panel merging data from the different INGI cohorts, using the method implemented by the IMPUTE2 software. The data were then added to the 1000G Phase 3 reference panel to obtain the IGRP1.0 reference.

**4. Reference panel quality assessment**

We assessed the quality of the addition of our sequence data to the 1000G Phase 3 imputation reference panel in terms of genotype concordance (r^2^) and information score (info score) metrics provided by the IMPUTE2 software. First, we removed all monomorphic sites since they don’t add any information in term of “usefulness” for further genetic analyses after imputation and stratified by minor allele frequency bins as follow: (0 - 0.005], (0.005 - 0.01], (0.01 - 0.02], (0.02 - 0.05], (0.05 - 0.1], (0.1 - 0.2], (0.2 - 0.5] (upper boundaries included in each interval). To compare r^2^ performances, only genotyped sites for each frequency bin were selected, and a Wilcoxon rank sum test (one tail test - H1: results from IGRP1.0 based imputation yield higher average r^2^ values ) between data imputed with our IGRP1.0 panel and 1000G Phase3 panel was performed (Supplementary Table 4). A resampling test was also performed selecting a random sample of 100 sites for each frequency bin, and repeating the Wilcoxon test for 1000 times: the estimated p-value for the randomisation method was computed with the equation:

P =$\frac{1+\sum_{j=1}^{B} I({x_{j}\geq X)}}{B+1}$

where X represents the value of the statistic calculated for the whole set, B the number of replicates, x_j_ the value of the statistic calculated for the current replicate j, I is the function that assumes value of 1 if the current replicate statistic is as large as the one for the real data and 0 otherwise [13].

We applied the Wilcoxon rank sum test to examine differences in the distribution of info score between the IGRP1.0 and 1000G Phase3 panels. In addition to the MAF binning we also defined five INFO score bins: (0 - 0.2], (0.2 - 0.4], (0.4 - 0.6], (0.6 - 0.8], (0.8 - 1]. The Wilcoxon rank sum test was applied to each info score bin in each minor allele frequency bin (one tail test - H1: results from IGRP1.0 based imputation yield higher average info score values ). **Supplementary Table 5** shows the results for comparisons in the rare variant bin (MAF <= 0.5%). A thousand random replicates were generated using the minimum number of imputed sites represented in one of the info score bins as resampling size for each info score bin in each MAF bin. The estimated p-value was computed with the formula described above.

Randomisation tests results showed for r^2^ and info score, in each population tested a p-value of 9.99x10^-4^.

**5. Genome-Wide Association Studies (GWAS)**

Genomic kinship was used to take into account the relatedness in the INGI cohorts. Variants with significant p-value (<0.05) for heterogeneity Cochran Q were excluded from the meta-analysis in addition to filtering applied by MAF, info score and effect direction. Bonferroni correction thresholds applied were P=6.23x10^-9^ for 1000G and P=4.69x10^-9^ for IGRP1.0.

For the 1000G imputation results, novel associations were identified for MCH and MCV both on chromosome 11 at 3.8 Mb (GRCh37 chr11:g.3820252C>T - rs117802349, MCH P=2.67x10^-10^, MCV P=2.33x10^-11^) and two variants on chromosome 11 at 5.2 and 5.4 Mb were identified for MCV, near beta-globin cluster (GRCh37 chr11:g.5263771G>C - rs113853911 - P=4.01x10^-9^ and GRCh37 chr11:g.5488177C>T - rs80297185, P=1.84x10^-14^ - D’ = 1, r^2^=0.328). For low frequency variants (MAF<1%), significant results were obtained on chromosome 2 (MCH, P=9.44x10^-10^), 5 (GRCh37 chr5:g.119073612A>G - rs142048030, MCH P=1.98x10^-10^ and MCV P=1.16x10^-9^), and 8 (GRCh37 chr8:g.125262331C>T - rs112483810, MCV P=3.86x10^-10^). As shown in **Supplementary Figure 5**, lambdas of GWAS meta-analysis with IGRP1.0 imputation were higher than lambda values of 1000G imputation, due to the high number of rare variants included in the new panel. However, the values ranged from 1.032 (MCHC) to 1.050 (RBC), indicating adequate control of population stratification. The meta-analysis of results of IGRP1.0 imputed data showed several GWAS significant results, mainly in low frequency and rare variants (**Supplementary Table 10**). The best hits for HGB, MCH, MCV, and RBC were found in HBB cluster (chr11p15.4). The IGRP1.0 meta-analysis for HGB, MCH, MCV, and RBC identified the pathogenic SNP rs11549407 (GRCh37 chr11:g.5248004G>A) located in HBB gene and responsible of beta-thalassemia and also associated with lipid traits in Sardinian populations (MCV P=1.86x10^-59^, MCH P=4.88x10^-52^, RBC P=8.30x10^-14^, HGB P=3.67x10^-10^) [14]. This SNP was discovered with higher p-values (using IGRP1.0 respect to 1000G) for MCHC (P=0.0001) and HCT (P=5.38x10^-7^). This locus was found only in CAR and VBI, and this rare variant (CAR MAF= 0.48% and VBI MAF= 0.28%) was present neither in FVG nor in 1000G EUR. Furthermore, this variant has a low frequency, as reported in Exac European (AF=0.07%) and an r^2^ value of 0.328 (D’=1) with the rs113853911 variant identified in the previous analysis using 1000G and discovered also with IGRP1.0 (only in HGB and HCT with p-values of 1.68x10^-4^ and 2.49x10^-4^ respectively).

**6. Population Structure**

Specific commands used for the population structure analyses are reported. Runs of homozygosity (ROH) and inbreeding coefficient were estimated using PLINK with the command --homozyg and --het on a total of 6 144 500 markers using the following filters: --maf 0.01 --hwe 0.00000001 --homozyg --homozyg-kb 1000 --homozyg-snp 50 --homozyg-gap 1000 --homozyg-density 50 --homozyg-window-het 1 --homozyg-window-threshold 0.05 --homozyg-window-missing 5.

The analysis of ancestry components performed with ADMIXTURE v1.2 used the European population plus one African reference (YRI) one East Asian (CHB) and one South Asian (GIH). Cross-validation error procedure was implemented for cluster solution between 2 and 12.

**7. The pattern of Natural Selection in Italian Populations**

We selected genes comprising candidate markers in only one population (|iHS|≥2 in one population but not in the others) and markers with |iHS|≥2 in all Italian populations. We required at least 20 markers with |iHS|≥2 overlapping a gene region, in order to be considered under putative selection. Here we report the main results for the genes with IHS cutoff of 2. A total of 37 genes were found under putative selection in all Italian populations (**Supplementary Table 12**). Interestingly, six genes (*FHIT*, *CSMD1*, *CNTNAP2*, *MACROD2*, *RBFOX1* and *PTPRD*) were found under putative selection in all Italian populations but with different markers. Some of them had been previously associated with complex traits such as *FHIT* associated with BMI [15], *CSMD1* associated with 79 different phenotypes, including age of menarche [16], schizophrenia [17] and educational attainment [18] (data from GWAS catalogue), *CNTNAP2* associated with mathematical ability [18] and DNA methylation variation [19], *MACROD2* associated with several phenotypes, including educational attainment [18] and blood protein levels [20], *RBFOX1* associated with eyes [21], other neurological traits and also educational attainment [18] and *PTPRD* associated with restless leg syndrome [22] and blood pressure [23].

To provide some examples of the complexity of the pattern of selection signatures in the Italian peninsula we selected the highest-ranking genes in terms of |iHS| and number of SNPs with |iHS|>=2 that are found only in one population. In the current Italian reference (TSI), we found a strong signal for *TYW1B*, associated with triglycerides [24] and educational attainment [18]. We found a signature in *CYP2C19* in CAR associated with diastolic blood pressure [25], *ABCG8* in VBI associated with lipid traits [26] , *SLC25A12* in SMC (educational attainment [18]), *ERI3* in CLZ (educational attainment [18]). In ERT we found marked signatures for *ANKRD30A*, which was associated with paediatric autoimmune diseases, metabolite levels and vestibular neuritis [27], in SAU we found evidence for the *CLOCK* gene associated with height [28], we found *SSPN* in ILG (associated with atrial fibrillation [29]) and *PBRM1* in RSI which was associated with blood protein levels [20], schizophrenia, general cognitive ability [30].

Using a more stringent criterion (|iHS|>=2.5), we discovered a total of 375 genes under selection. We discovered 29 different genes with high iHS in all Italian cohorts, a full list of the result with |iHs|>2.5 is reported in **Supplementary Table 13**. With this selection criterion, the populations with the highest number of private signals are TSI (60 genes) and RSI (63 genes).

These are only examples of the different genomic patterns that can be found in the various subpopulations of the Italian peninsula. The hypothesis here that the private signals of selection are indeed the results of it, but in some isolates, such signals were lost due to bottleneck and demography. Nevertheless, we cannot exclude that some results are indeed private of a specific population.

**8. Human Knockout**

Loss of Function (LoF) variants represent a category of deleterious variants, whose homozygous state could lead to human KO (HKO), more in detail the LoF are frameshift, splice acceptor variant, splice donor variant, stop gained, stop lost, start lost, transcript ablation, transcript amplification. Besides, considering that the highest enrichment of deleterious variants in our populations was found for variants with CADD score ≥20, we used this value to select LoF variation. In our total cohort, we found 506 LoF presenting with a CADD≥20 at homozygous state in at least one individual per population (**Supplementary Table 15**). Gene ontology analysis revealed an excess of transmembrane signalling receptor genes, including olfactory receptors, as already described. In order to have a high reliable dataset, we used stringent filtering criteria and analysed only variants that affected all transcripts (considered as TOTAL LoF in opposition to PARTIAL LoF), resulting in 205 variants affecting 195 different genes **.** Among these 205 variants, the majority (150, ~73%) was shared among all three cohorts, and nearly 60% had frequency ≥ 0.05**.** A large number of HKOs was located in genes involved in hair/skin/epithelium or eye phenotypes, and many were members of gene families. Specifically, five HKOs were found in keratin genes: (*KRT37*, *KRT24*, *KRT31* and *KRT83*) and 5 in keratin associated protein genes (*KRTAP1-5*, *KRTAP1-1*, *KRTAP19-6* and *KRTAP13-2*, *KRTAP29-1*). Two different rare stop gain variants were found in *KRT83* (MAF in Europeans GRCh37 chr12:g.52711747C>A - rs146753414 - is 0.027, GRCh37 chr12:g.52711549G>A - rs2857667 - is 0.006). Two missense variants in this gene are associated with a mild form of monilethrix (MNLIX; OMIM #158000), a rare autosomal dominant hair disease that results in fragile, brittle hair that tends to fracture and produce some degree of alopecia [31]. The hair of three carriers of the KO of *KRT83* in the VBI population and of nine heterozygotes in three families was investigated and resulted normal. The lack of KRT83 does not seem to affect hair structure as much as substitutions of amino acids that are highly conserved and affect the helix termination motifs, known hotspots for monilethrix mutations. Finally, we found a very rare stop gain (GRCh37 chr3:g.130190723delT - rs11355796) in *COL6A5* gene (collagen type VI, alpha 5) identified in a homozygous state in one individual from VBI. The variant is at higher frequency in all three Italian populations (AF~0.015 compared with reference Europeans of 0.0013). Variants in the *COL6A5* gene were shown to cause familial neuropathic chronic itch [32]. Unfortunately, we do not possess clinical data on our HKO so far, but we were able to interview a related heterozygous carrier, who reported to suffer from itching all his life. We then investigated the role of natural selection on HKO: the expectation is that HKO could arise in genes where the selection pressure is less effective or where there is no selection [33]. Among the whole LoF set (TOTAL and PARTIAL), we analysed only variants reported in gnomAD (to apply more stringent filtering and avoid the chance of false positives), overlapping 133 different genes, which are distributed among the populations as shown in **Supplementary Figure 15**. We found that the majority of genes in which HKO were detected are unique of FVG, VBI and CAR (61, 36 and 10 respectively) whereas only 13 genes are shared among all populations. Among these HKO genes, only two show evidence of selection in the same population in which the HKO carriers are present (see **Supplementary Table 16**) confirming the expectation. In addition the majority of the RVIS score (residual variation intolerance score) for the whole set of HKO genes are positive (median=0.73) and significant different (Wilcoxon–Mann–Whitney P=7x10^-55^) from the whole set of genes reported (median=-0.05), which indicates an overall relaxation of purifying selection in our HKO list.

**9. Data availability**

A subset of the data is already available on the European Genome-phenome Archive (EGA) at the following links:

FVG cohort: BAM files <https://www.ebi.ac.uk/ega/studies/EGAS00001000252>; sample list, vcf files <https://www.ebi.ac.uk/ega/studies/EGAS00001001597> ; <https://www.ebi.ac.uk/ega/datasets/EGAD00001002729> ;

VBI cohort: BAM files <https://www.ebi.ac.uk/ega/studies/EGAS00001000398>; <https://www.ebi.ac.uk/ega/studies/EGAS00001000458>;

CAR cohort: BAM files <https://www.ebi.ac.uk/ega/studies/EGAS00001000460>;

A vcf file including all the INGI variants (SNPs and INDELs) with information on allele frequencies in the whole dataset and each cohort, has been submitted to the European Variation Archive (EVA), study accession number: PRJEB33648. The data is accessible at the following link: <https://www.ebi.ac.uk/ena/data/view/PRJEB33648>.

**Supplementary references:**

1. BridgeBuilder efficiently remaps BAM/SAM reads to a new reference by first building a &quot;bridge&quot; reference, first mapping to that bridge, and then remapping only a subset of reads to the fu.. [Internet]. Wellcome Trust Sanger Institute - Human Genetics Informatics; 2015 [cited 2019 Jan 3]. Available from: https://github.com/wtsi-hgi/bridgebuilder

2. https://broadinstitute.github.io/picard/. Picard Tools - By Broad Institute [Internet]. [cited 2016 Dec 14]. Available from: https://broadinstitute.github.io/picard/

3. McKenna A, Hanna M, Banks E, Sivachenko A, Cibulskis K, Kernytsky A, et al. The Genome Analysis Toolkit: a MapReduce framework for analyzing next-generation DNA sequencing data. Genome Res. 2010 Sep;20(9):1297–303.

4. Li H. A statistical framework for SNP calling, mutation discovery, association mapping and population genetical parameter estimation from sequencing data. Bioinforma Oxf Engl. 2011 Nov 1;27(21):2987–93.

5. DePristo MA, Banks E, Poplin R, Garimella KV, Maguire JR, Hartl C, et al. A framework for variation discovery and genotyping using next-generation DNA sequencing data. Nat Genet. 2011 May;43(5):491–8.

6. 1000 Genomes Project Consortium, Abecasis GR, Auton A, Brooks LD, DePristo MA, Durbin RM, et al. An integrated map of genetic variation from 1,092 human genomes. Nature. 2012 Nov 1;491(7422):56–65.

7. Browning SR, Browning BL. Rapid and Accurate Haplotype Phasing and Missing-Data Inference for Whole-Genome Association Studies By Use of Localized Haplotype Clustering. Am J Hum Genet. 2007 Jan 11;81(5):1084–97.

8. Delaneau O, Howie B, Cox AJ, Zagury J-F, Marchini J. Haplotype Estimation Using Sequencing Reads. Am J Hum Genet. 2013 Oct 3;93(4):687–96.

9. Howie BN, Donnelly P, Marchini J. A Flexible and Accurate Genotype Imputation Method for the Next Generation of Genome-Wide Association Studies. PLoS Genet. 2009 Jun 19;5(6):e1000529.

10. Sudmant PH, Rausch T, Gardner EJ, Handsaker RE, Abyzov A, Huddleston J, et al. An integrated map of structural variation in 2,504 human genomes. Nature. 2015 Oct 1;526(7571):75–81.

11. Sherry ST, Ward MH, Kholodov M, Baker J, Phan L, Smigielski EM, et al. dbSNP: the NCBI database of genetic variation. Nucleic Acids Res. 2001 Jan 1;29(1):308–11.

12. McLaren W, Pritchard B, Rios D, Chen Y, Flicek P, Cunningham F. Deriving the consequences of genomic variants with the Ensembl API and SNP Effect Predictor. Bioinforma Oxf Engl. 2010 Aug 15;26(16):2069–70.

13. Ruxton GD, Neuhäuser M. Improving the reporting of P-values generated by randomization methods. Methods Ecol Evol. 2013;4(11):1033–6.

14. Danjou F, Zoledziewska M, Sidore C, Steri M, Busonero F, Maschio A, et al. Genome-wide association analyses based on whole-genome sequencing in Sardinia provide insights into regulation of hemoglobin levels. Nat Genet [Internet]. 2015 Sep 14 [cited 2015 Oct 2];advance online publication. Available from: http://www.nature.com/ng/journal/vaop/ncurrent/full/ng.3307.html

15. Hoffmann TJ, Choquet H, Yin J, Banda Y, Kvale MN, Glymour M, et al. A Large Multiethnic Genome-Wide Association Study of Adult Body Mass Index Identifies Novel Loci. Genetics. 2018 Oct 1;210(2):499–515.

16. Perry JRB, Day F, Elks CE, Sulem P, Thompson DJ, Ferreira T, et al. Parent-of-origin-specific allelic associations among 106 genomic loci for age at menarche. Nature. 2014 Oct;514(7520):92–7.

17. Bergen SE, O’Dushlaine CT, Ripke S, Lee PH, Ruderfer DM, Akterin S, et al. Genome-wide association study in a Swedish population yields support for greater CNV and MHC involvement in schizophrenia compared with bipolar disorder. Mol Psychiatry. 2012 Sep;17(9):880–6.

18. Lee JJ, Wedow R, Okbay A, Kong E, Maghzian O, Zacher M, et al. Gene discovery and polygenic prediction from a genome-wide association study of educational attainment in 1.1 million individuals. Nat Genet. 2018 Aug;50(8):1112.

19. Zhang Q, Marioni RE, Robinson MR, Higham J, Sproul D, Wray NR, et al. Genotype effects contribute to variation in longitudinal methylome patterns in older people. Genome Med. 2018 Oct 22;10(1):75.

20. Sun BB, Maranville JC, Peters JE, Stacey D, Staley JR, Blackshaw J, et al. Genomic atlas of the human plasma proteome. Nature. 2018 Jun;558(7708):73.

21. Pickrell JK, Berisa T, Liu JZ, Ségurel L, Tung JY, Hinds DA. Detection and interpretation of shared genetic influences on 42 human traits. Nat Genet [Internet]. 2016 May 16 [cited 2016 May 26];advance online publication. Available from: http://www.nature.com/ng/journal/vaop/ncurrent/full/ng.3570.html

22. Schormair B, Zhao C, Bell S, Tilch E, Salminen AV, Pütz B, et al. Identification of novel risk loci for restless legs syndrome in genome-wide association studies in individuals of European ancestry: a meta-analysis. Lancet Neurol. 2017 Nov 1;16(11):898–907.

23. Evangelou E, Warren HR, Mosen-Ansorena D, Mifsud B, Pazoki R, Gao H, et al. Genetic analysis of over 1 million people identifies 535 new loci associated with blood pressure traits. Nat Genet. 2018 Oct;50(10):1412.

24. Teslovich TM, Musunuru K, Smith AV, Edmondson AC, Stylianou IM, Koseki M, et al. Biological, clinical and population relevance of 95 loci for blood lipids. Nature. 2010 Aug;466(7307):707–13.

25. Liu C, Kraja AT, Smith JA, Brody JA, Franceschini N, Bis JC, et al. Meta-analysis identifies common and rare variants influencing blood pressure and overlapping with metabolic trait loci. Nat Genet. 2016 Oct;48(10):1162–70.

26. Chasman DI, Paré G, Mora S, Hopewell JC, Peloso G, Clarke R, et al. Forty-Three Loci Associated with Plasma Lipoprotein Size, Concentration, and Cholesterol Content in Genome-Wide Analysis. PLOS Genet. 2009 Nov 20;5(11):e1000730.

27. Li YR, Li J, Zhao SD, Bradfield JP, Mentch FD, Maggadottir SM, et al. Meta-analysis of shared genetic architecture across ten pediatric autoimmune diseases. Nat Med. 2015 Sep;21(9):1018–27.

28. Wood AR, Esko T, Yang J, Vedantam S, Pers TH, Gustafsson S, et al. Defining the role of common variation in the genomic and biological architecture of adult human height. Nat Genet. 2014 Nov;46(11):1173–86.

29. Nielsen JB, Thorolfsdottir RB, Fritsche LG, Zhou W, Skov MW, Graham SE, et al. Biobank-driven genomic discovery yields new insight into atrial fibrillation biology. Nat Genet. 2018 Sep;50(9):1234.

30. Davies G, Lam M, Harris SE, Trampush JW, Luciano M, Hill WD, et al. Study of 300,486 individuals identifies 148 independent genetic loci influencing general cognitive function. Nat Commun. 2018 May 29;9(1):2098.

31. Steensel MAM van, Steijlen PM, Bladergroen RS, Vermeer M, Geel M van. A missense mutation in the type II hair keratin hHb3 is associated with monilethrix. J Med Genet. 2005 Mar 1;42(3):e19–e19.

32. Martinelli-Boneschi F, Colombi M, Castori M, Devigili G, Eleopra R, Malik RA, et al. COL6A5 variants in familial neuropathic chronic itch. Brain. 2017 Mar 1;140(3):555–67.

33. Alkuraya FS. Human knockout research: new horizons and opportunities. Trends Genet. 2015 Feb 1;31(2):108–15.
