# Supplementary material for: A bird’s-eye view of Italian genomic variation through whole-genome sequencing
Source: Eur J Hum Genet. 2019 Nov 29;28(4):435–44. doi: 10.1038/s41431-019-0551-x (PMC7080768; doi:10.1038/s41431-019-0551-x)
Supplement: Supplementary file 3 — Supplementary Table 1 [file 41431_2019_551_MOESM3_ESM.docx]

**Supplementary table 1.**The proportion of sites shared and not shared with outbred populations. All data are aligned to the Human genome reference build 37 (GRCh37).

| **INGI PRIVATE** | | | | | | |
| --- | --- | --- | --- | --- | --- | --- |
|  | **1000GPh3** | **INGI/**  **1000GPh3** | **EUR-1000GPh3** | **INGI/EUR** | **UK10K+**  **1000GPh3** | **INGI/UK10K+**  **1000GPh3** |
| **Sites** | 9 973 892 | 37.47% | 12 144 189 | 45.62% | 9 120 634 | 34.26% |
| **Multiallelic Sites** | 241 653 | 43.08% | 255 310 | 45.52% | 299 571 | 53.41% |
| **SNPs** | 9 154 172 | 37.28% | 11 259 369 | 45.85% | 8 392 468 | 34.17% |
| **INDELs** | 819 720 | 39.76% | 884 820 | 42.92% | 728 166 | 35.32% |
| **Singletons SNPs** | 4 262 732 | 68.83% | 5 203 426 | 84.01% | 3 959 423 | 63.93% |
| **Singletons INDELs** | 211 862 | 77.41% | 235 902 | 86.20% | 186 931 | 68.30% |
| **Sites proportion** | | | | | | |
| **MAF <= 1%** | 9 186 091 | 92.10% | 11 341 870 | 93.39% | 8 344 618 | 91.49% |
| **1% < MAF <= 5%** | 390 473 | 3.91% | 403 919 | 3.33% | 399 176 | 4.38% |
| **MAF > 5%** | 526 492 | 5.28% | 531 574 | 4.38% | 539 536 | 5.92% |
|  |  |  |  |  |  |  |
| **INGI SHARED** | | | | | | |
|  | **1000GPh3** | **INGI/**  **1000GPh3** | **EUR-1000GPh3** | **INGI/EUR** | **UK10K+**  **1000GPh3** | **INGI/UK10K+**  **1000GPh3** |
| **Sites** | 16 645 199 | 62.53% | 14 474 902 | 54.38% | 17 498 457 | 65.74% |
| **Multiallelic Sites** | 319 265 | 56.92% | 305 608 | 54.48% | 261 347 | 46.59% |
| **SNPs** | 15 403 194 | 62.72% | 13 297 997 | 54.15% | 16 164 898 | 65.83% |
| **INDELs** | 1 242 005 | 60.24% | 1 176 905 | 57.08% | 1 333 559 | 64.68% |
| **Singletons SNPs** | 1 930 754 | 31.17% | 990 060 | 15.99% | 2 234 063 | 36.07% |
| **Singletons INDELs** | 61 817 | 22.59% | 37 777 | 13.80% | 86 748 | 31.70% |
| **Sites proportion** | | | | | | |
| **MAF <= 1%** | 7 499 860 | 45.06% | 5 344 081 | 36.92% | 8 341 333 | 47.67% |
| **1% < MAF <= 5%** | 2 735 498 | 16.43% | 2 722 052 | 18.81% | 2 726 795 | 15.58% |
| **MAF > 5%** | 6 596 572 | 39.63% | 6 591 490 | 45.54% | 6 583 528 | 37.62% |
