# Supplementary material for: A bird’s-eye view of Italian genomic variation through whole-genome sequencing
Source: Eur J Hum Genet. 2019 Nov 29;28(4):435–44. doi: 10.1038/s41431-019-0551-x (PMC7080768; doi:10.1038/s41431-019-0551-x)
Supplement: Supplementary file 4 — Supplementary Table 2 [file 41431_2019_551_MOESM4_ESM.docx]

**Supplementary Table 2.** **Variants selected from each INGI cohort for the creation of the reference panel.** The table shows the number of sites passing each step of filtering. The final set used for the IGRP1.0 panel creation retained 95.6% 94.29% and 92.06% of the WGS variants for CAR, FVG and VBI respectively. All data are aligned to the Human genome reference build 37 (GRCh37).

|  | **AC >=2 and DP >=5** | **Singletons overlapping with 1000GPh3 or UK10K** | **Singletons overlapping at least between 2 INGI cohors** | **TOTAL** |
| --- | --- | --- | --- | --- |
| **SNPs** | | | | |
| **VBI** | 14 269 966 | 1 079 579 | 639 777 | **15 989 322** |
| **FVG** | 12 750 969 | 801 378 | 704 408 | **14 256 755** |
| **CAR** | 10 072 498 | 462 609 | 874 559 | **11 409 666** |
|  |  |  |  |  |
| **INDELs** | | | | |
| **VBI** | 1 778 080 | 36 801 | 20 104 | **1 834 985** |
| **FVG** | 1 711 655 | 36 254 | 27 318 | **1 775 227** |
| **CAR** | 1 318 278 | 19 636 | 35 310 | **1 373 224** |
