# Supplementary material for: A bird’s-eye view of Italian genomic variation through whole-genome sequencing
Source: Eur J Hum Genet. 2019 Nov 29;28(4):435–44. doi: 10.1038/s41431-019-0551-x (PMC7080768; doi:10.1038/s41431-019-0551-x)
Supplement: Supplementary file 5 — Supplementary Table 3 [file 41431_2019_551_MOESM5_ESM.docx]

**Supplementary Table 3**: **Sites added to the 1000GP phase 3 reference.**

The table shows the number of sites obtained merging the INGI WGS data with 1000GP phase 3 reference panel: 7.8% of IGRP1.0 panel variants are Italian population specific. All data are aligned to the Human genome reference build 37 (GRCh37).

|  | **Sites INGI** | **Sites INGI+TGP3** | **Sites TGP3** | **Sites added by INGI** |
| --- | --- | --- | --- | --- |
| **SNPs** | 20 824 903 | 83 963 965 | 78 397 635 | 5 566 330 |
| **INDELs** | 2 510 222 | 4 645 443 | 3 308 387 | 1 337 056 |
| **tot** | 23 335 125 | 88 609 408 | 81 706 022 | 6 903 386 |
