# Supplementary material for: A bird’s-eye view of Italian genomic variation through whole-genome sequencing
Source: Eur J Hum Genet. 2019 Nov 29;28(4):435–44. doi: 10.1038/s41431-019-0551-x (PMC7080768; doi:10.1038/s41431-019-0551-x)
Supplement: Supplementary file 6 — Supplementary Table 4 [file 41431_2019_551_MOESM6_ESM.docx]

**Supplementary Table 4:** Comparison of r^2^ score performances on genotyped sites for each frequency bin. The p-values from the Wilcoxon rank sum test (one tail test - H1: results from IGRP1.0 based imputation yield higher average r^2^ score values) are shown. All data are aligned to the Human genome reference build 37 (GRCh37).

| **MAF** | **CAR** | **FVG** | **VBI** | **NW-ITALY** | **KORCULA** | **SPLIT** | **VIS** |
| --- | --- | --- | --- | --- | --- | --- | --- |
| **<= 0.5%** | 0.7000 | 7.06E-04 | 1.48E-03 | 0.975 | 0.379 | 0.715 | 0.500 |
| **0.5% - 1%** | 0.6432 | 6.18E-05 | 4.09E-04 | 0.741 | 0.331 | 0.495 | 0.424 |
| **1% - 2%** | 0.1213 | 5.28E-04 | 1.17E-03 | 0.660 | 0.359 | 0.493 | 0.386 |
| **2% - 5%** | 0.0450 | 5.78E-08 | 5.69E-06 | 0.441 | 0.420 | 0.333 | 0.161 |
