# Supplementary material for: A bird’s-eye view of Italian genomic variation through whole-genome sequencing
Source: Eur J Hum Genet. 2019 Nov 29;28(4):435–44. doi: 10.1038/s41431-019-0551-x (PMC7080768; doi:10.1038/s41431-019-0551-x)
Supplement: Supplementary file 7 — Supplementary Table 5 [file 41431_2019_551_MOESM7_ESM.docx]

**Supplementary Table 5:** Number of variants imputed for each panel in the lowest frequency bin stratified by INFO score. Wilcoxon test p-values between IGRP1.0 and 1000G imputation are reported (one tail test - H1: results from IGRP1.0 based imputation yield higher average info score values per bin). All data are aligned to the Human genome reference build 37 (GRCh37).

|  |  | **CAR** | | | |  |
| --- | --- | --- | --- | --- | --- | --- |
|  |  | **INGI+TGP3** | | **TGP3** | |  |
| **MAF** | **INFO BIN** | **N sites** | **%** | **N sites** | **%** | **p-value** |
| **<= 0.5%** | **0.2** | 384 735 | 35.27% | 411 773 | 41.69% | 0 |
|  | **0.4** | 237 605 | 21.78% | 205 986 | 20.86% | 4.75E-16 |
|  | **0.6** | 169 309 | 15.52% | 134 497 | 13.62% | 2.98E-08 |
|  | **0.8** | 138 947 | 12.74% | 106 712 | 10.80% | 5.84E-01 |
|  | **1** | 160 120 | 14.68% | 128 659 | 13.03% | 1 |
|  |  |  |  |  |  |  |
|  |  | **FVG** | | | |  |
|  |  | **INGI+TGP3** | | **TGP3** | |  |
| **MAF** | **INFO BIN** | **N sites** | **%** | **N sites** | **%** | **p-value** |
| **<= 0.5%** | **0.2** | 208 007 | 22.70% | 384 192 | 39.64% | 0 |
|  | **0.4** | 238 894 | 26.07% | 263 543 | 27.19% | 0 |
|  | **0.6** | 239 173 | 26.10% | 183 333 | 18.92% | 3.53E-191 |
|  | **0.8** | 230 332 | 25.13% | 138 085 | 14.25% | 1.53E-56 |
|  | **1** | 187 589 | 16.99% | 96 939 | 9.09% | 7.39E-08 |
|  |  |  |  |  |  |  |
|  |  | **VBI** | | | |  |
|  |  | **INGI+TGP3** | | **TGP3** | |  |
| **MAF** | **INFO BIN** | **N sites** | **%** | **N sites** | **%** | **p-value** |
| **<= 0.5%** | **0.2** | 180 980 | 15.92% | 309 013 | 30.06% | 6.29E-227 |
|  | **0.4** | 211 835 | 18.63% | 241 107 | 23.46% | 4.39E-277 |
|  | **0.6** | 224 152 | 19.72% | 180 750 | 17.58% | 2.09E-237 |
|  | **0.8** | 242 050 | 21.29% | 148 491 | 14.45% | 7.17E-119 |
|  | **1** | 277 924 | 24.44% | 148 525 | 14.45% | 1 |
|  |  |  |  |  |  |  |
|  |  | **NW-ITA** | | | |  |
|  |  | **INGI+TGP3** | | **TGP3** | |  |
| **MAF** | **INFO BIN** | **N sites** | **%** | **N sites** | % | **p-value** |
| **<= 0.5%** | **0.2** | 313 018 | 21.77% | 254 779 | 21.34% | 8.91E-119 |
|  | **0.4** | 323 960 | 22.53% | 241 114 | 20.19% | 7.04E-06 |
|  | **0.6** | 279 392 | 19.43% | 204 901 | 17.16% | 1 |
|  | **0.8** | 246 843 | 17.17% | 216 962 | 18.17% | 1 |
|  | **1** | 274 486 | 19.09% | 276 277 | 23.14% | 9.77E-39 |

|  |  | **KORCULA** | | | |  |
| --- | --- | --- | --- | --- | --- | --- |
|  |  | **INGI+TGP3** | | **TGP3** | |  |
| **MAF** | **INFO BIN** | **N sites** | **%** | **N sites** | % | **p-value** |
| **<= 0.5%** | **0.2** | 308,323 | 29.32% | 241,440 | 29.95% | 2.32E-285 |
|  | **0.4** | 269,317 | 25.61% | 178,187 | 22.10% | 1.77E-04 |
|  | **0.6** | 191,300 | 18.19% | 134,773 | 16.72% | 1 |
|  | **0.8** | 129,230 | 12.29% | 102,975 | 12.77% | 1 |
|  | **1** | 153,443 | 14.59% | 148,828 | 18.46% | 1 |
|  |  |  |  |  |  |  |
|  |  | **SPLIT** | | | |  |
|  |  | **INGI+TGP3** | | **TGP3** | |  |
| **MAF** | **INFO BIN** | **N sites** | **%** | **N sites** | **%** | **p-value** |
| **<= 0.5%** | **0.2** | 263,410 | 30.48% | 266,992 | 29.48% | 3.41E-73 |
|  | **0.4** | 199,252 | 23.05% | 192,622 | 21.27% | 4.71E-03 |
|  | **0.6** | 147,321 | 17.04% | 143,457 | 15.84% | 2.68E-01 |
|  | **0.8** | 121,059 | 14.01% | 129,660 | 14.32% | 1 |
|  | **1** | 133,266 | 15.42% | 172,920 | 19.09% | 1 |
|  |  |  |  |  |  |  |
|  |  | **VIS** | | | |  |
|  |  | **INGI+TGP3** | | **TGP3** | |  |
| **MAF** | **INFO BIN** | **N sites** | **%** | **N sites** | **%** | **p-value** |
| **<= 0.5%** | **0.2** | 405 658 | 33.60% | 336 034 | 34.31% | 4.40E-160 |
|  | **0.4** | 303 034 | 25.10% | 223 648 | 22.83% | 2.01E-03 |
|  | **0.6** | 210 856 | 17.46% | 160 484 | 16.38% | 1 |
|  | **0.8** | 151 656 | 12.56% | 128 354 | 13.10% | 1 |
|  | **1** | 136 226 | 11.28% | 130 995 | 13.37% | 9.95E-01 |
