# Supplementary material for: A bird’s-eye view of Italian genomic variation through whole-genome sequencing
Source: Eur J Hum Genet. 2019 Nov 29;28(4):435–44. doi: 10.1038/s41431-019-0551-x (PMC7080768; doi:10.1038/s41431-019-0551-x)
Supplement: Supplementary file 8 — Supplementary Table 6 [file 41431_2019_551_MOESM8_ESM.docx]

**Supplementary Table 6.** Rare variants IGRP1.0 imputation panel performance. All data are aligned to the Human genome reference build 37 (GRCh37).

| **MAF<0.5%**  **Info score>=0.4** | **N sites TGP3** | **Nistes IGRP 1.0** | **% of sites well imputed in TGP3** | **% of sites well imputed in IGRP 1.0** |
| --- | --- | --- | --- | --- |
| **FVG** | 418 357 | 657 094 | 39.24% | 59.52% |
| **VBI** | 477 766 | 744 126 | 46.48% | 65.45% |
| **CAR** | 369 868 | 468 376 | 37.45% | 42.94% |
| **NW-ITA** | 698 140 | 800 721 | 58.47% | 55.69% |
| **KORCULA** | 386 576 | 473 973 | 47.95% | 45.07% |
| **VIS** | 419 833 | 498 738 | 42.86% | 41.31% |
| **SPLIT** | 446 037 | 541 302 | 49.25% | 47.18% |
