# Supplementary material for: A bird’s-eye view of Italian genomic variation through whole-genome sequencing
Source: Eur J Hum Genet. 2019 Nov 29;28(4):435–44. doi: 10.1038/s41431-019-0551-x (PMC7080768; doi:10.1038/s41431-019-0551-x)
Supplement: Supplementary file 9 — Supplementary Table 7 [file 41431_2019_551_MOESM9_ESM.docx]

**Supplementary Table 7:** comparison between variants retained in the IGRP1.0 belonging from INGI only cohorts but removed from the Haplotype consortium reference panel. All data are aligned to the Human genome reference build 37 (GRCh37).

|  | **All INGI only sites** | | | **MAF< 0.5%** | | |
| --- | --- | --- | --- | --- | --- | --- |
|  | **#sites** | **INFO score >= 0.4** | **%** | **#sites** | **INFO score >= 0.4** | **%** |
| **CAR** | 639 046 | 437 505 | 68.46% | 256 222 | 94 588 | 36.92% |
| **FVG** | 653 500 | 514 770 | 78.77% | 270 924 | 157 828 | 58.26% |
| **VBI** | 673 071 | 548 758 | 81.53% | 284 666 | 181 647 | 63.81% |
| **NW-ITALY** | 696 895 | 475 806 | 68.28% | 326 076 | 153 571 | 47.10% |
| **KORCULA** | 624 434 | 417 776 | 66.90% | 262 773 | 85 994 | 32.73% |
| **VIS** | 644 736 | 406 698 | 63.08% | 285 926 | 53 662 | 18.77% |
| **SPLIT** | 648 071 | 442 750 | 68.32% | 281 119 | 44 295 | 15.76% |
