# Supplementary material for: A bird’s-eye view of Italian genomic variation through whole-genome sequencing
Source: Eur J Hum Genet. 2019 Nov 29;28(4):435–44. doi: 10.1038/s41431-019-0551-x (PMC7080768; doi:10.1038/s41431-019-0551-x)
Supplement: Supplementary file 10 — Supplementary Table 8 [file 41431_2019_551_MOESM10_ESM.docx]

**Supplementary Table 8:** Characteristics of samples used for GWAS analyses. HGB = haemoglobin concentration, RBC = red blood cell count, HCT = haematocrit, MCH = mean corpuscular haemoglobin, MCHC = MCH concentration, MCV = mean corpuscular volume. The values are mean (standard deviation).

|  | **CAR** | **FVG** | **VBI** |
| --- | --- | --- | --- |
| N.individuals | 438 | 1262 | 1592 |
| Females (%) | 60.32 | 56.98 | 55.63 |
| Age, years | 49.8 (16.0) | 52.2 (16.5) | 54.7 (18.3) |
| HGB, g/dL | 13.9 (1.4) | 14.0 (1.3) | 14.4 (1.4) |
| MCHC, g/dL | 33.4 (1.5) | 33.0 (1.0) | 33.0 (1.6) |
| RBC, 1M cell/cmm | 4.77 (0.47) | 4.73 (0.43) | 4.82 (0.45) |
| HCT, % | 41.73 (3.97) | 42.5 (3.6) | 43.64 (3.74) |
| MCV, femtoliters | 87.8 (6.8) | 90.2 (4.1) | 90.8 (5.0) |
| MCH, picograms | 29.3 (2.9) | 29.8 (1.7) | 30 (1.9) |
