# Supplementary material for: A bird’s-eye view of Italian genomic variation through whole-genome sequencing
Source: Eur J Hum Genet. 2019 Nov 29;28(4):435–44. doi: 10.1038/s41431-019-0551-x (PMC7080768; doi:10.1038/s41431-019-0551-x)
Supplement: Supplementary file 16 — Supplementary Table 14 [file 41431_2019_551_MOESM16_ESM.docx]

**Supplementary Table 14:** LoF variants in a homozygous state in at least one individual in INGI cohorts. A variant was classified as TOTAL when it was predicted as LoF in all Ensembl database transcripts, otherwise, it was classified as PARTIAL. All data are aligned to the Human genome reference build 37 (GRCh37). VEP version 90 was used to determine each variant consequence.

|  | **Number of variants with CADD ≥ 20** | **Number of TOTAL LoF variants** | **Number of PARTIAL LoF variants** |
| --- | --- | --- | --- |
| **FRAMESHIFT** | 214 | 92 | 122 |
| **SPLICE ACCEPTOR** | 28 | 12 | 16 |
| **SPLICE DONOR** | 56 | 11 | 45 |
| **START LOST** | 19 | 7 | 12 |
| **STOP GAINED** | 185 | 82 | 103 |
| **STOP LOST** | 4 | 1 | 3 |
|  | 506 | 205 | 301 |
