# Supplementary material for: A bird’s-eye view of Italian genomic variation through whole-genome sequencing
Source: Eur J Hum Genet. 2019 Nov 29;28(4):435–44. doi: 10.1038/s41431-019-0551-x (PMC7080768; doi:10.1038/s41431-019-0551-x)
Supplement: Supplementary file 19 — Supplementary Table 17 [file 41431_2019_551_MOESM19_ESM.docx]

**Table Supplementary 17:** Number of sequenced samples from each sequencing centre in each cohort with mean coverage values.

| **Cohort** | **Sanger** | **BGI** | **HSR** |
| --- | --- | --- | --- |
| **Carlantino (CAR)** | 93 (4x) | 40 (10x) | 0 |
| **Friuli Venezia Giulia (FVG)** | 196 (4x) | 185 (10x) | 0 |
| **Val Borbera (VBI)** | 208 (6x) | 208 (6x) | 17 (6x) |
