# Supplementary material for: A bird’s-eye view of Italian genomic variation through whole-genome sequencing
Source: Eur J Hum Genet. 2019 Nov 29;28(4):435–44. doi: 10.1038/s41431-019-0551-x (PMC7080768; doi:10.1038/s41431-019-0551-x)
Supplement: Supplementary file 20 — Supplementary Table 18 [file 41431_2019_551_MOESM20_ESM.docx]

**Table Supplementary 18:** Non-Reference Discordance Rate (NRDR) average values calculated by site (**a**) and sample (**b**) comparing SNP array and Exome chip array to WGS data for each Italian cohort. All data are aligned to the Human genome reference build 37 (GRCh37).

| **a)** | **WGS** | | |
| --- | --- | --- | --- |
|  | **CAR** | **FVG** | **VBI** |
| **SNP array** | 2.94% | 0.46% | 0.82% |
| **Exome chip** | 2.47% | 0.45% | 0.72% |
|  |  |  |  |
| **b)** | **WGS** | | |
|  | **CAR** | **FVG** | **VBI** |
| **SNP array** | 2.024% | 0.77% | 0.68% |
| **Exome chip** | 2.25% | 4.23% | 0.72% |
