# Supplementary figures and images for: A bird’s-eye view of Italian genomic variation through whole-genome sequencing
Source: Eur J Hum Genet. 2019 Nov 29;28(4):435–44. doi: 10.1038/s41431-019-0551-x (PMC7080768; doi:10.1038/s41431-019-0551-x)

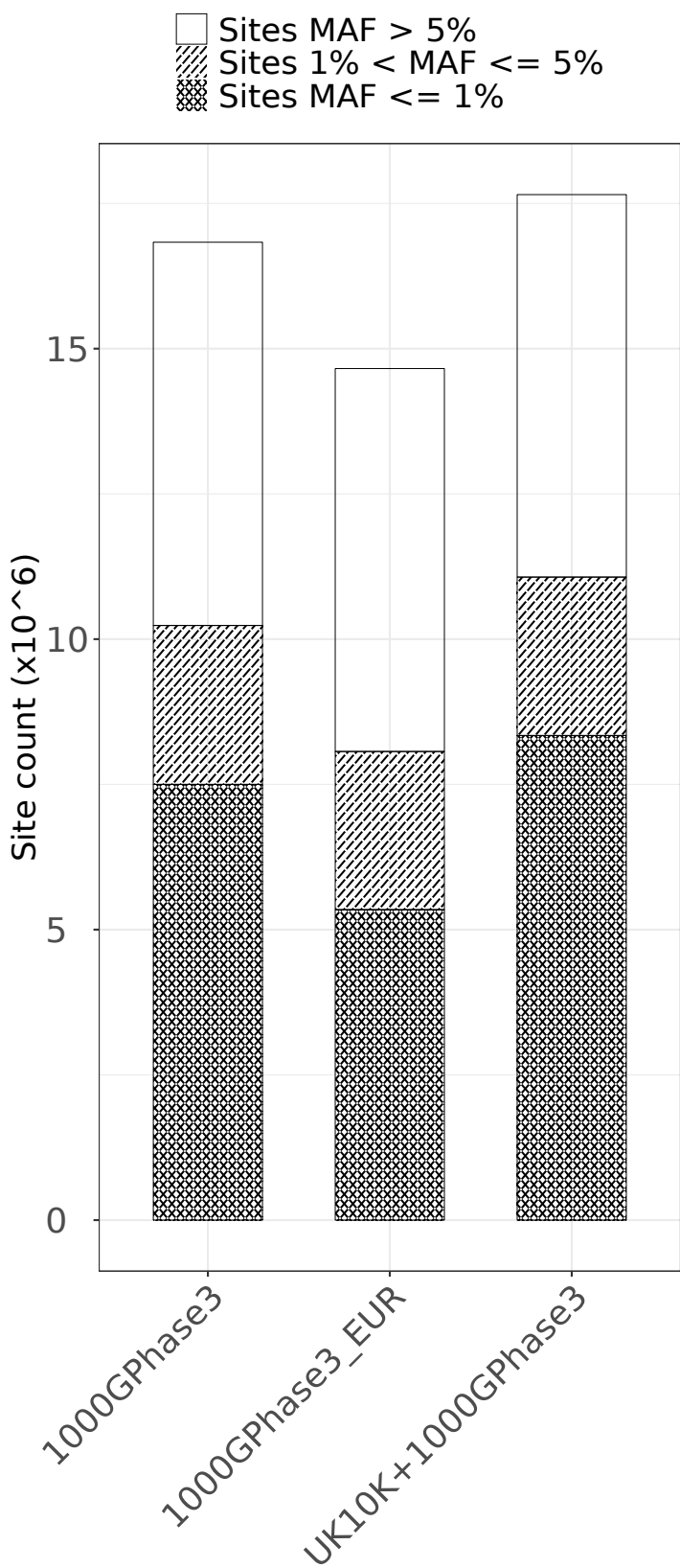

Supplementary Figure 1

Supplement: Supplementary file 21 — Supplementary Figure 1 [file 41431_2019_551_MOESM21_ESM.pdf]

Number of variants

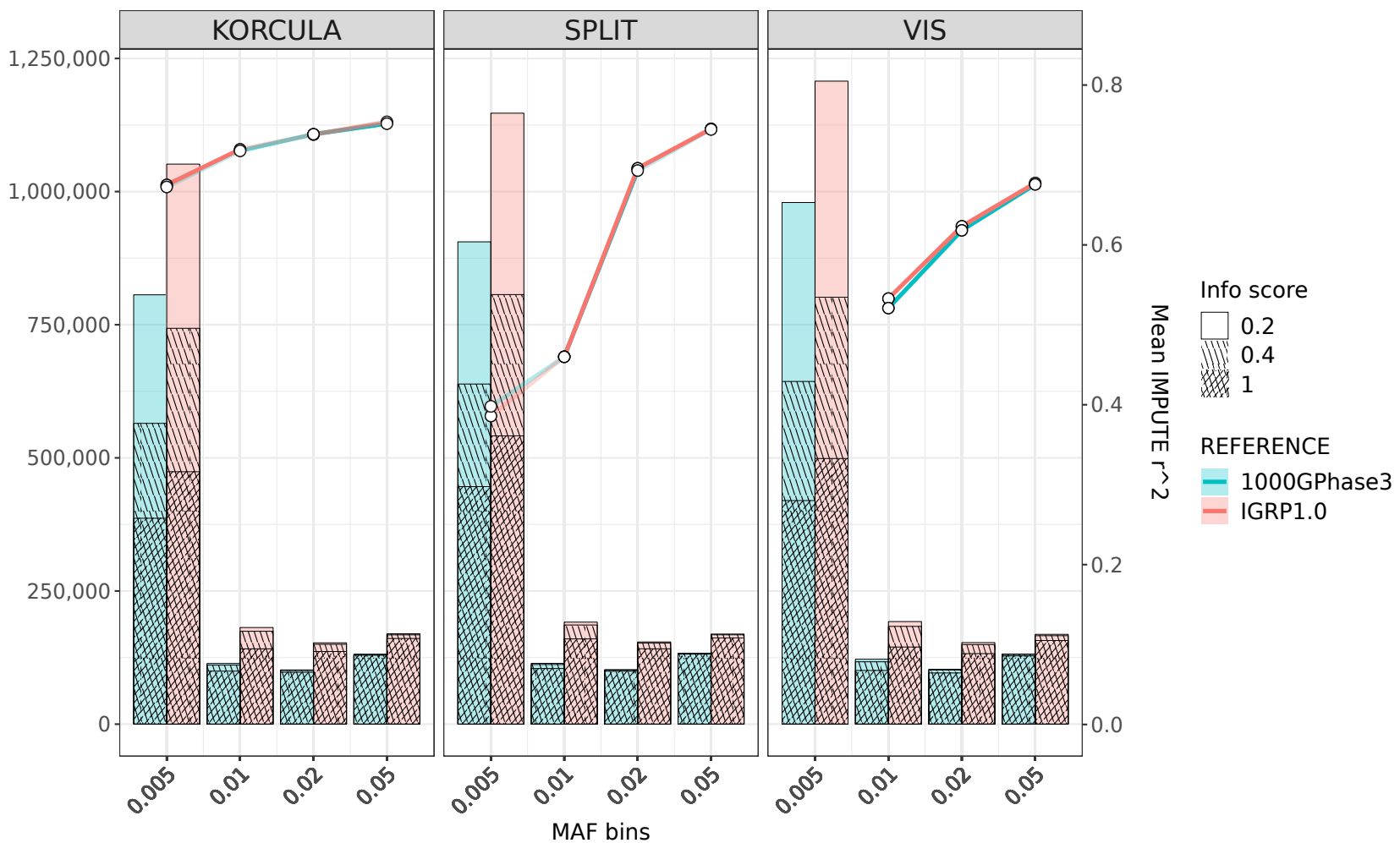

Supplementary Figure 2

Supplement: Supplementary file 22 — Supplementary Figure 2 [file 41431_2019_551_MOESM22_ESM.pdf]

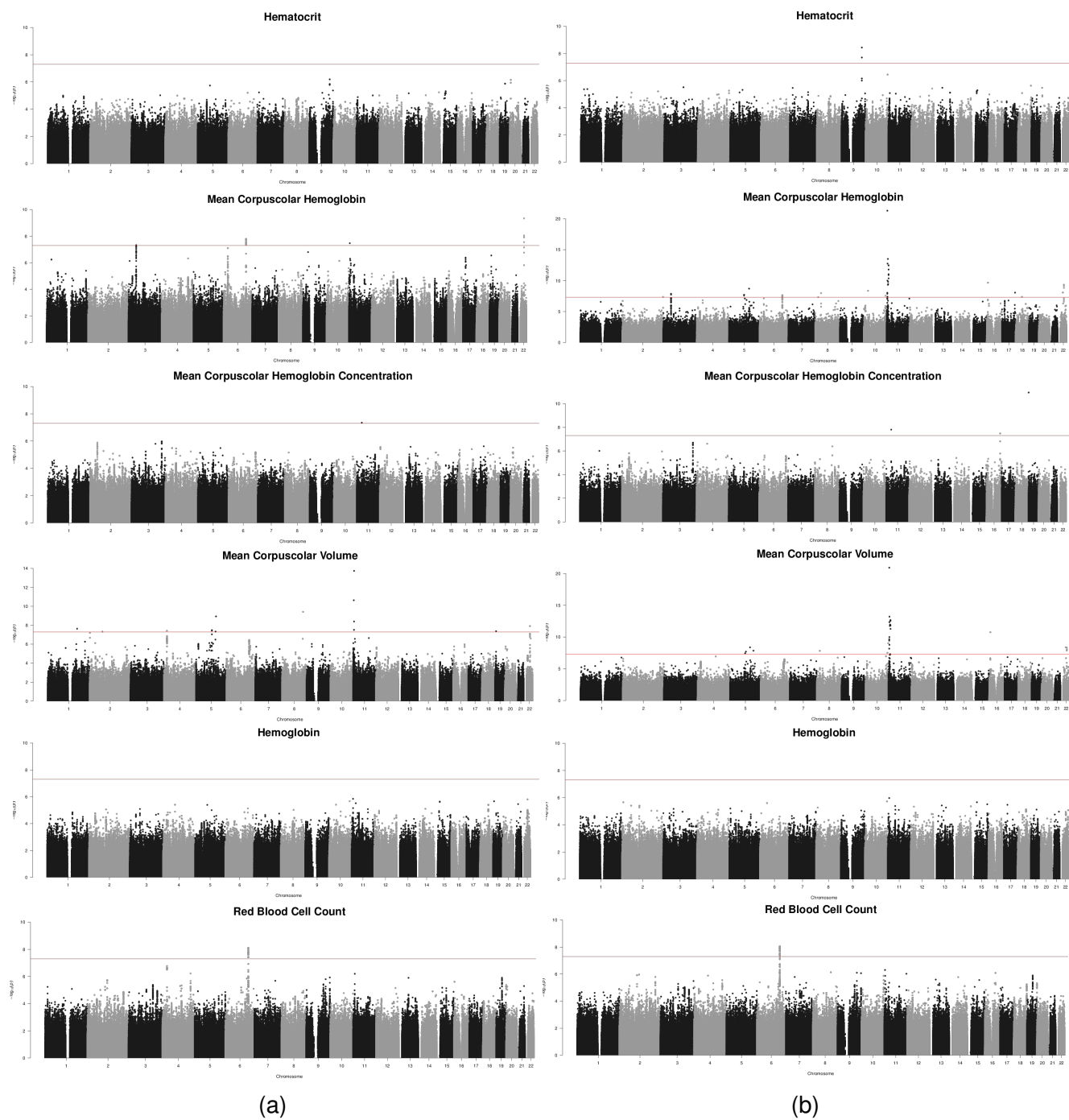

Supplementary Figure 3

Supplement: Supplementary file 23 — Supplementary Figure 3 [file 41431_2019_551_MOESM23_ESM.pdf]

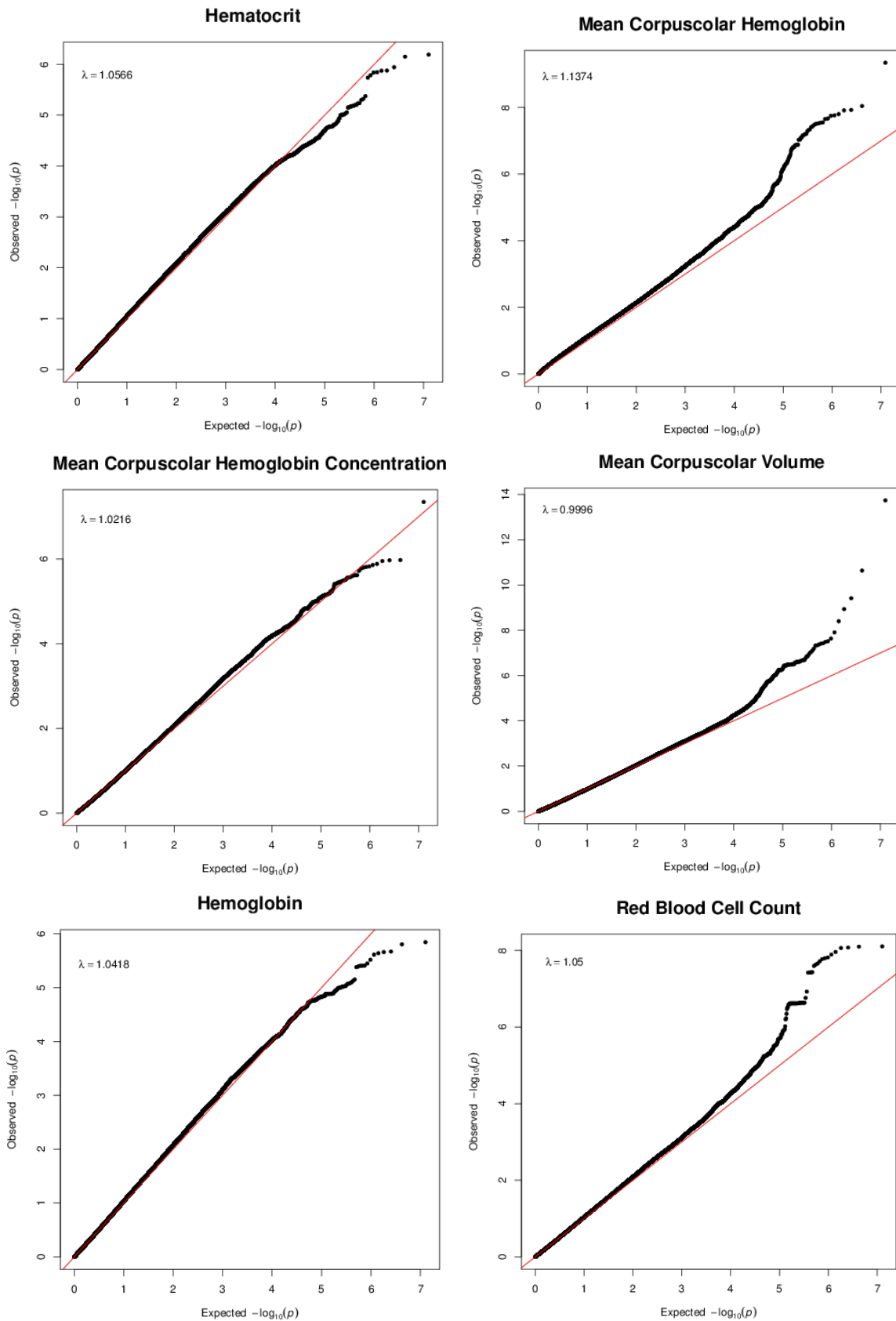

Supplementary Figure 4

Supplement: Supplementary file 24 — Supplementary Figure 4 [file 41431_2019_551_MOESM24_ESM.pdf]

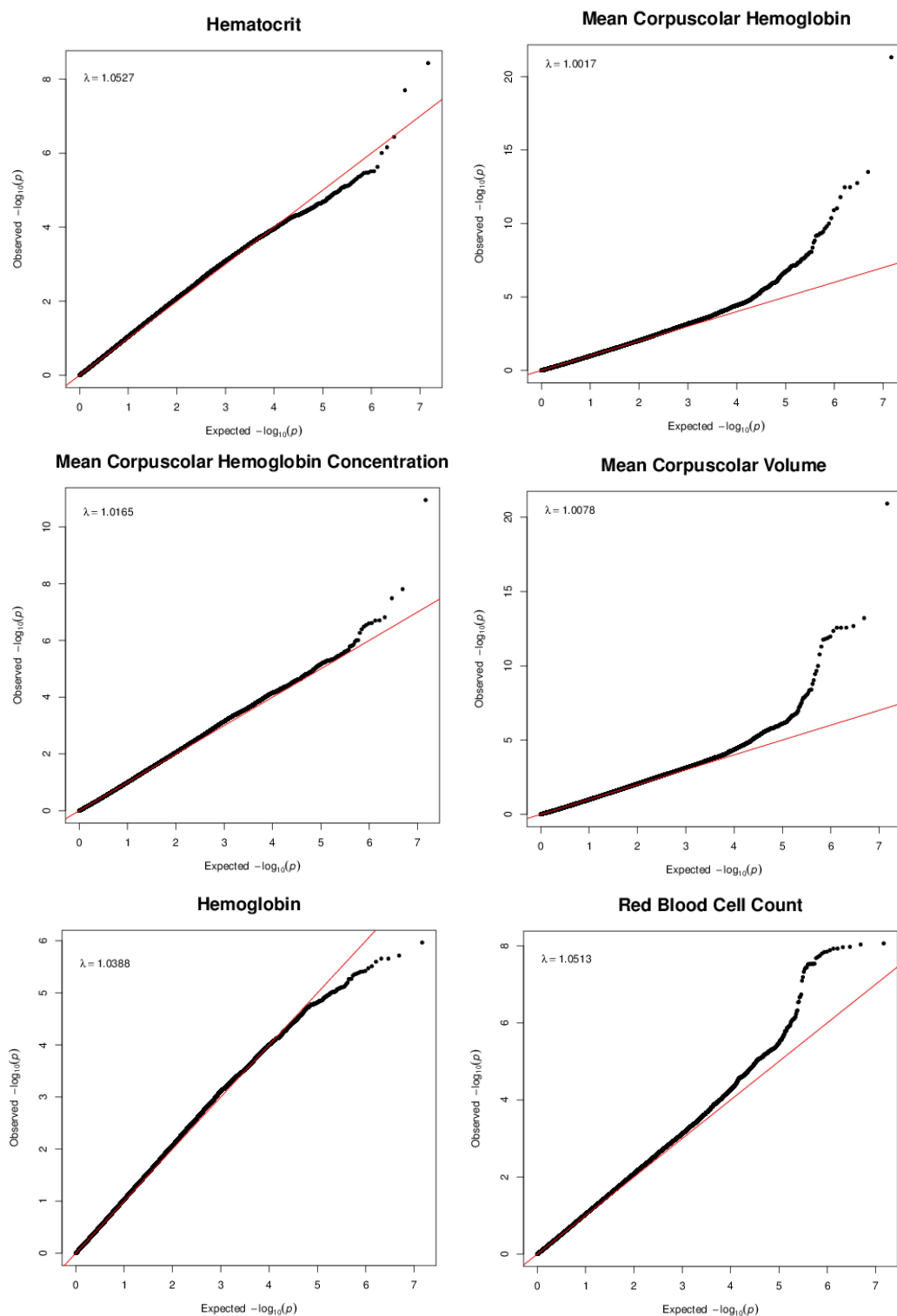

Supplementary Figure 5

Supplement: Supplementary file 25 — Supplementary Figure 5 [file 41431_2019_551_MOESM25_ESM.pdf]

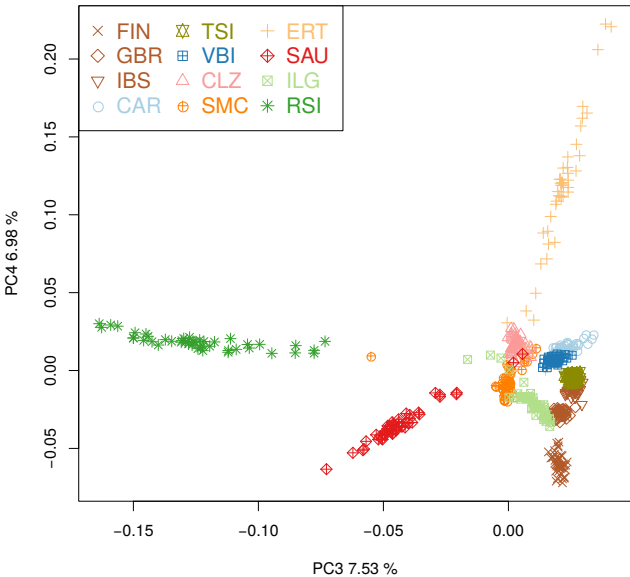

Supplementary Figure 6

Supplement: Supplementary file 26 — Supplementary Figure 6 [file 41431_2019_551_MOESM26_ESM.pdf]

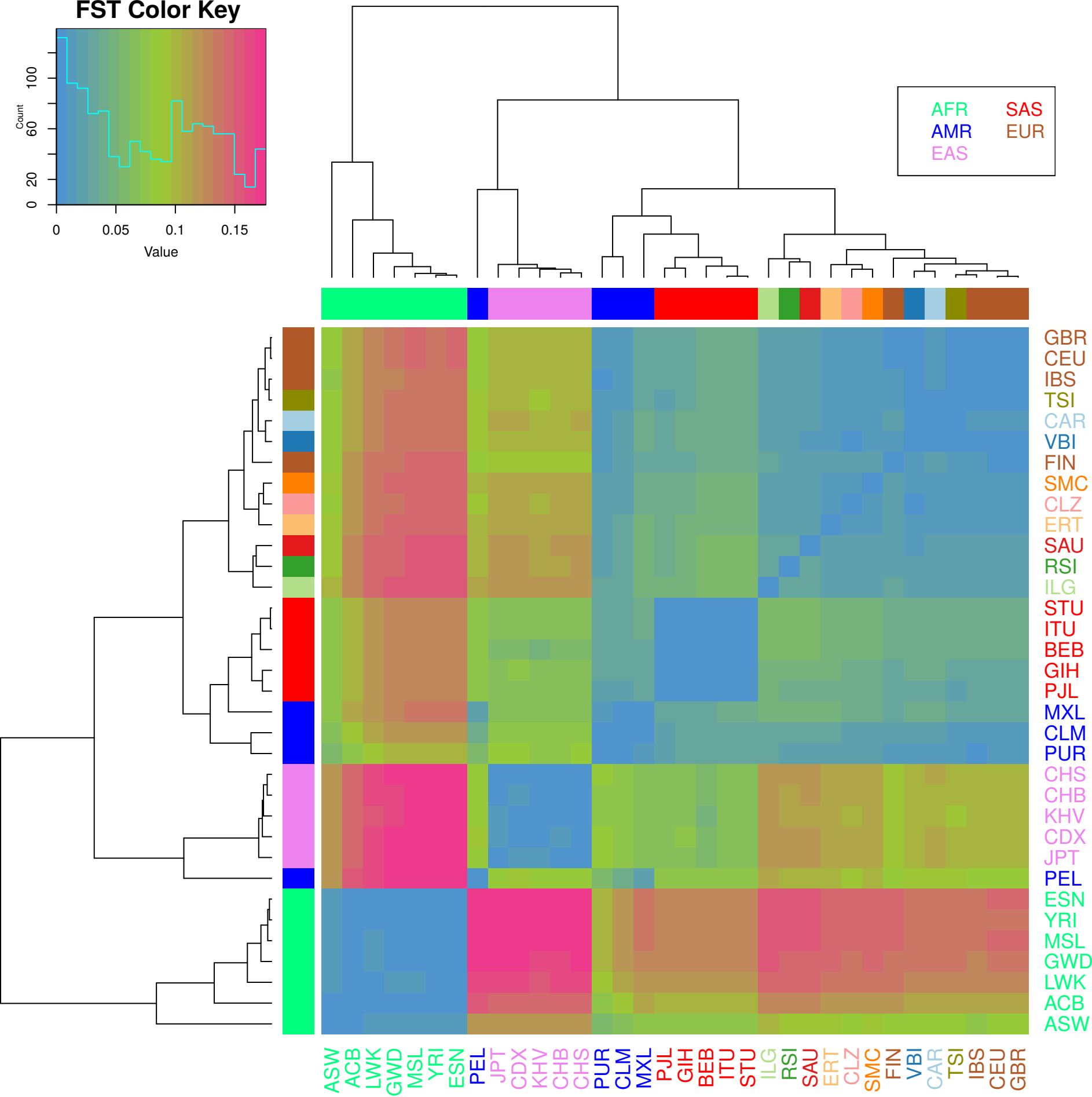

Supplementary Figure 7

Supplement: Supplementary file 27 — Supplementary Figure 7 [file 41431_2019_551_MOESM27_ESM.pdf]

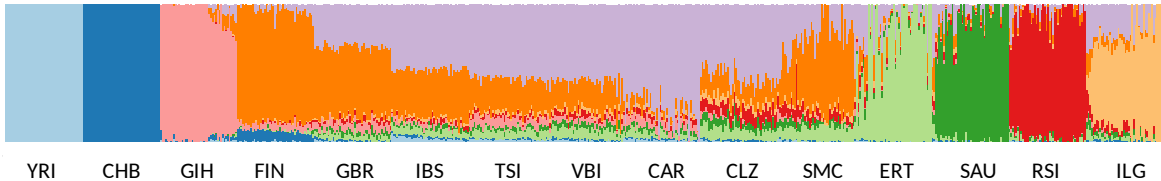

Supplementary Figure 8

Supplement: Supplementary file 28 — Supplementary Figure 8 [file 41431_2019_551_MOESM28_ESM.pdf]

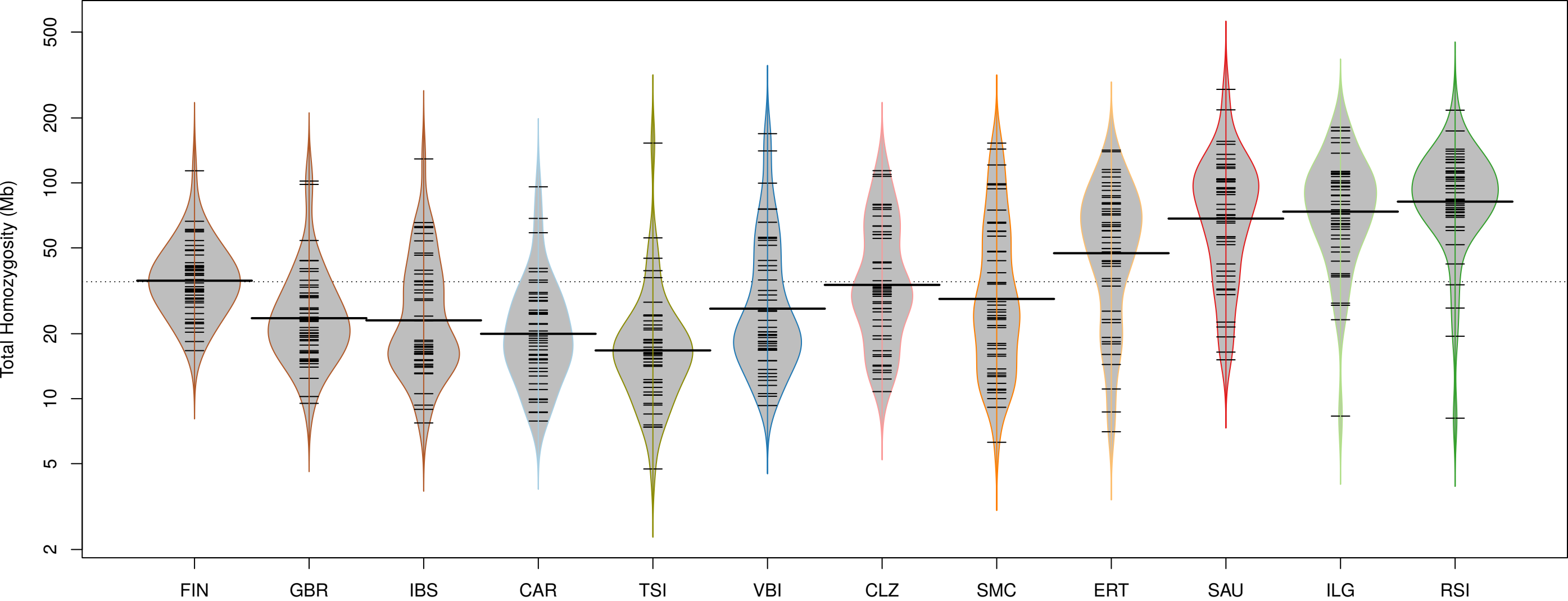

Supplementary Figure 9

Supplement: Supplementary file 29 — Supplementary Figure 9 [file 41431_2019_551_MOESM29_ESM.pdf]

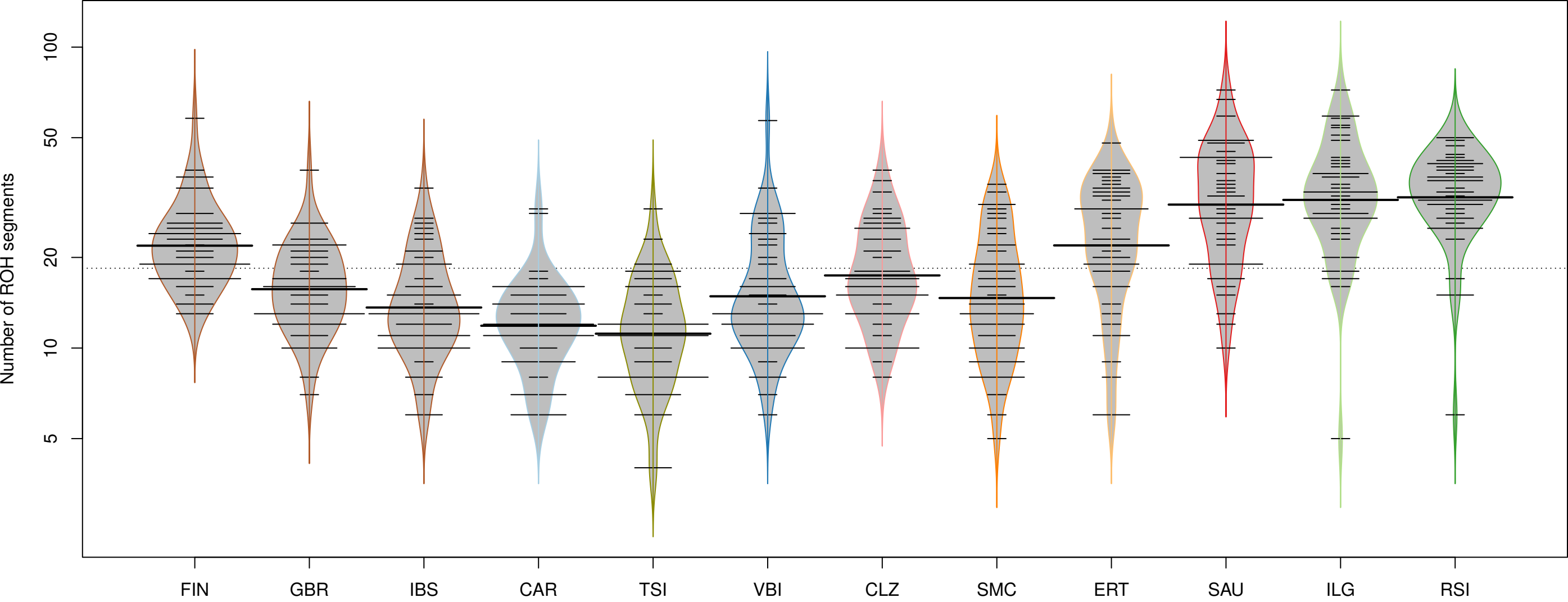

Supplementary Figure 10

Supplement: Supplementary file 30 — Supplementary Figure 10 [file 41431_2019_551_MOESM30_ESM.pdf]

## Genes under Selection $|iHS| \geq 2$

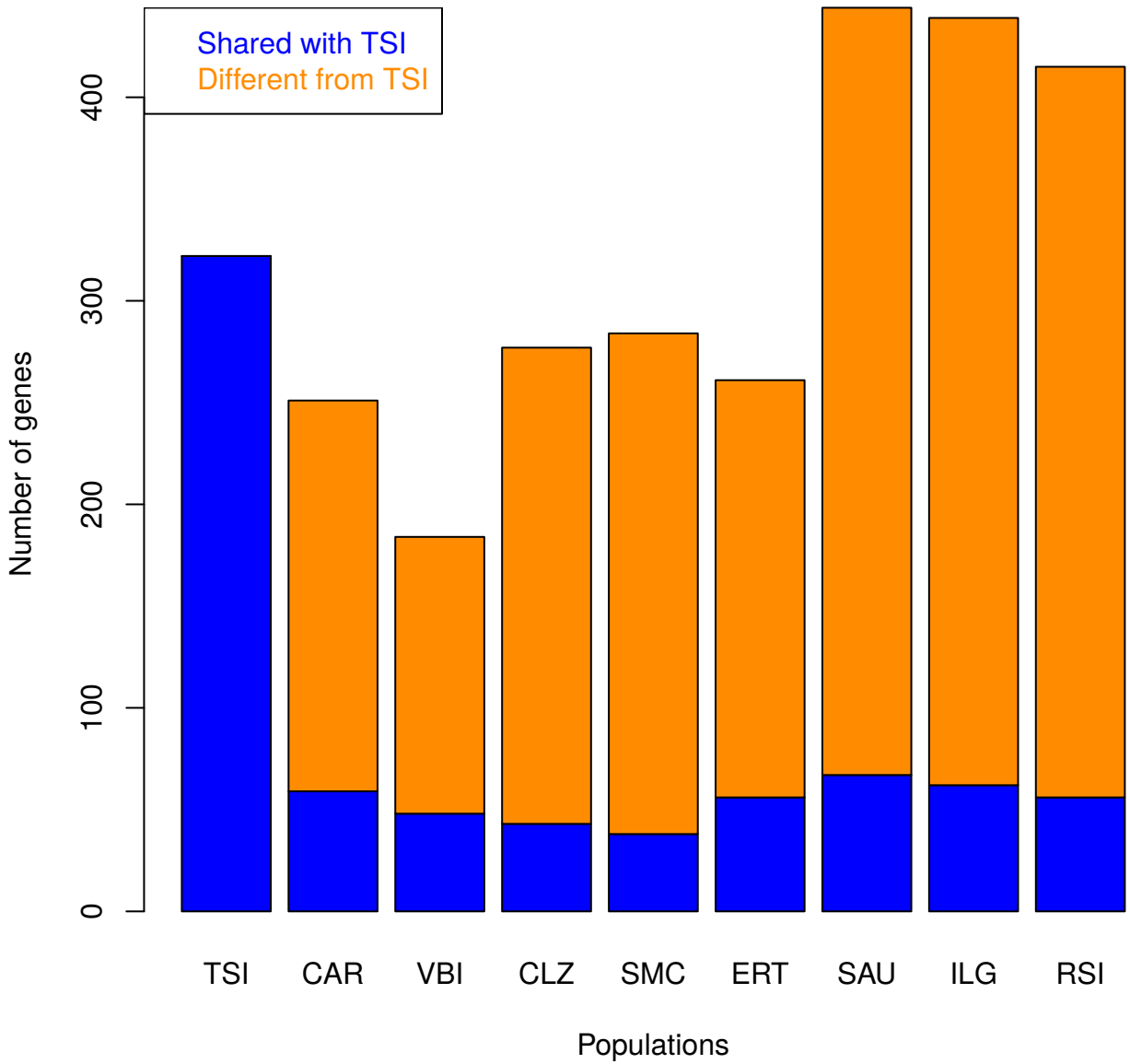

Supplement: Supplementary file 31 — Supplementary Figure 11 [file 41431_2019_551_MOESM31_ESM.pdf]

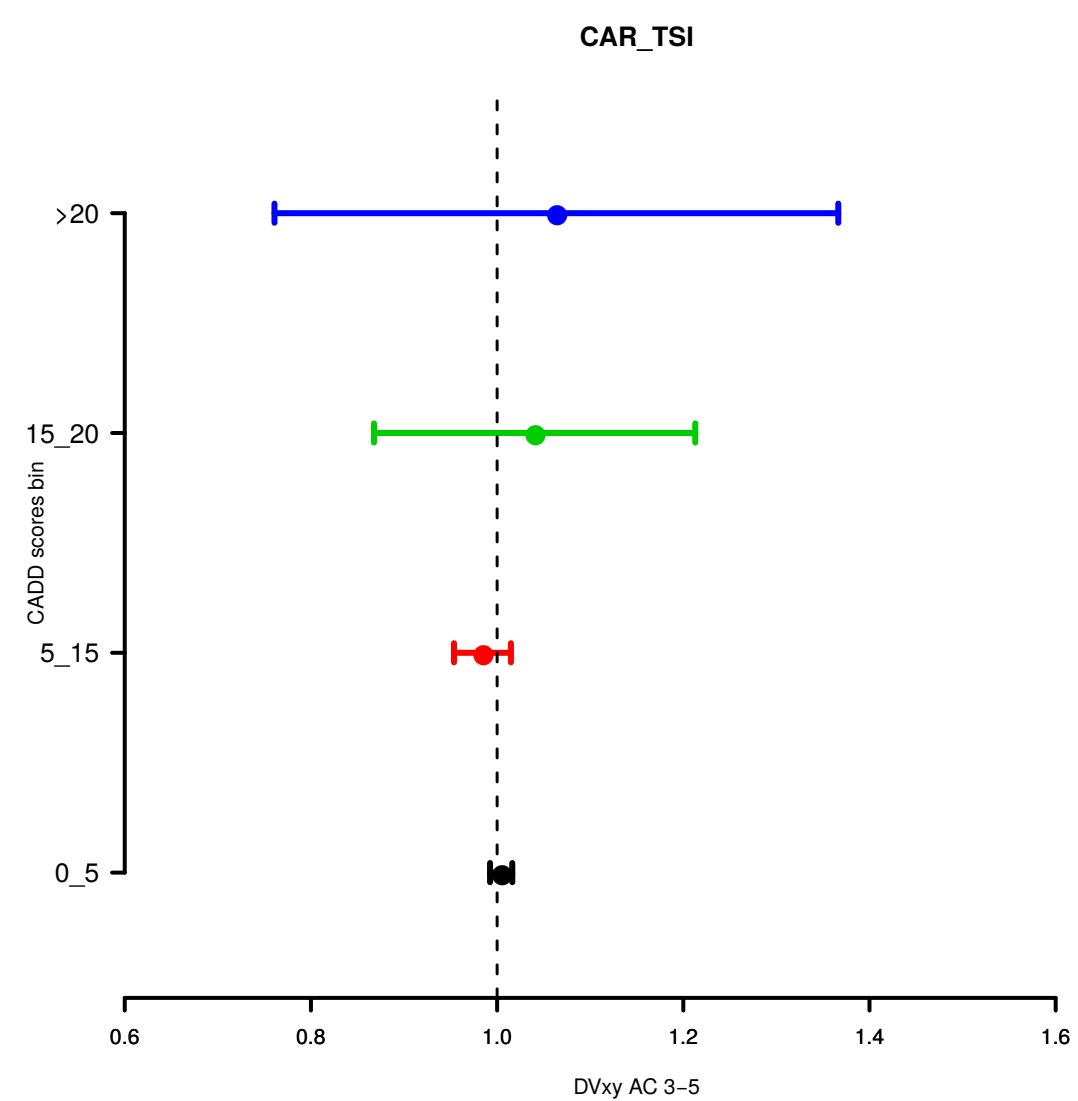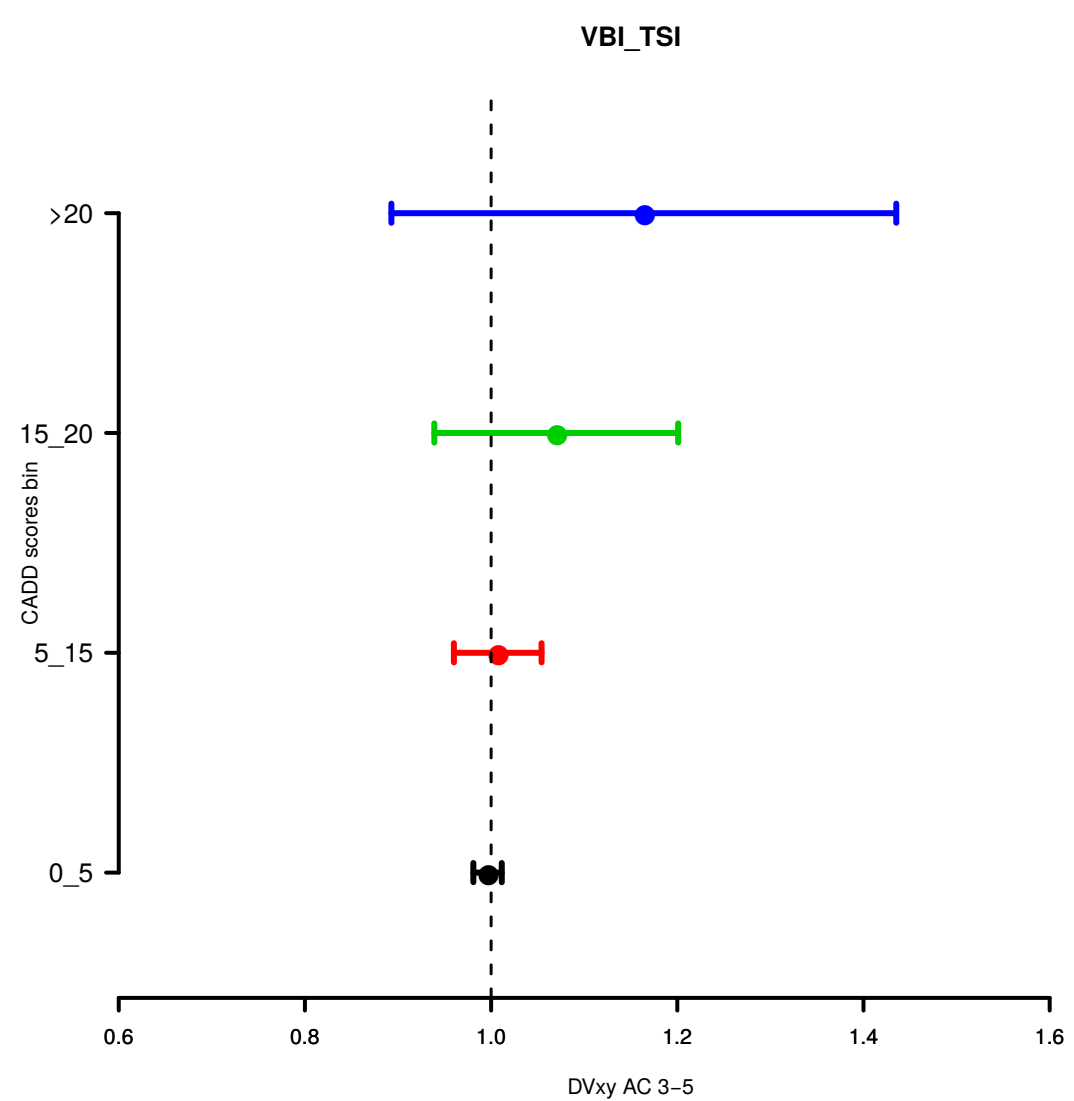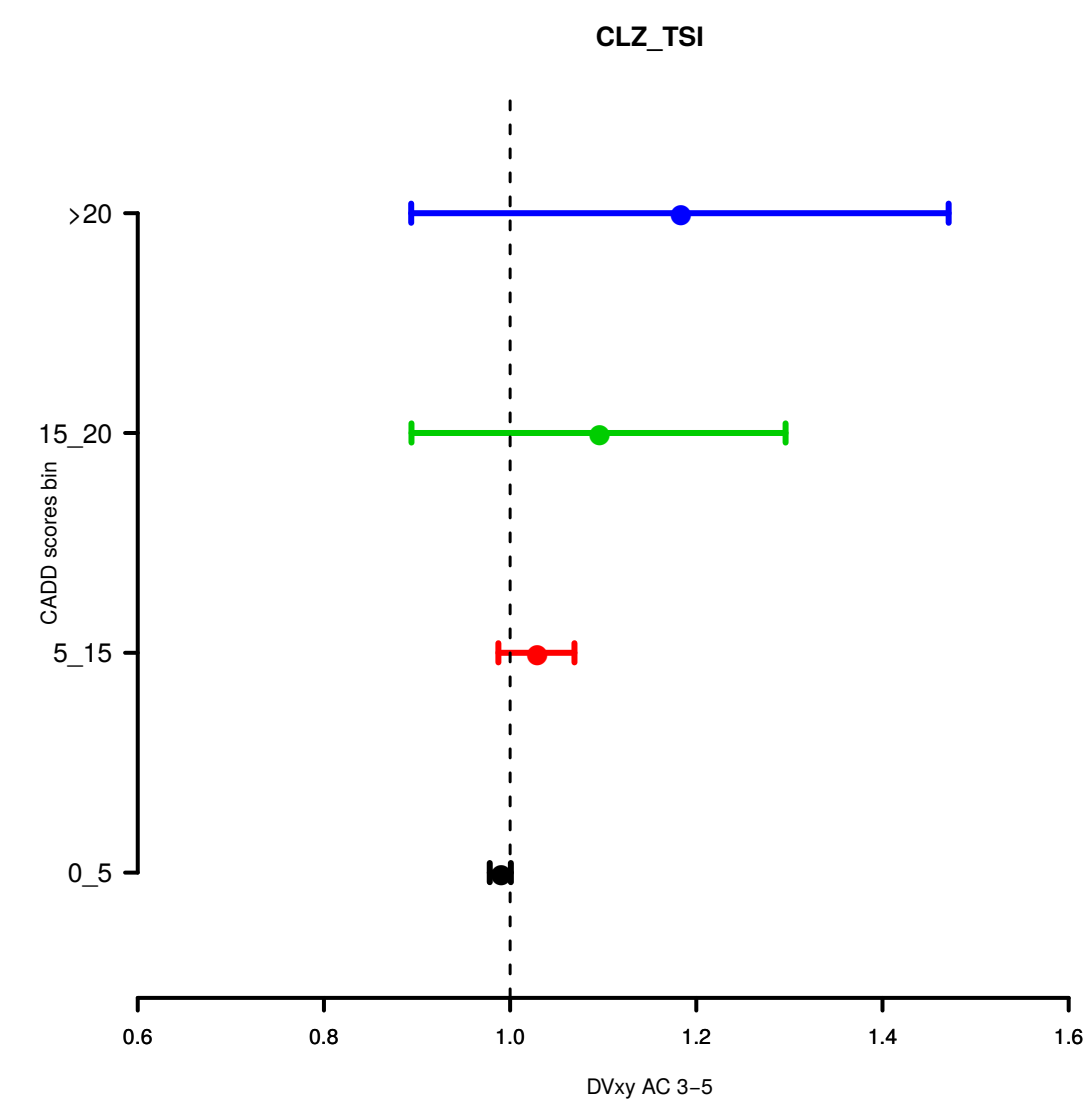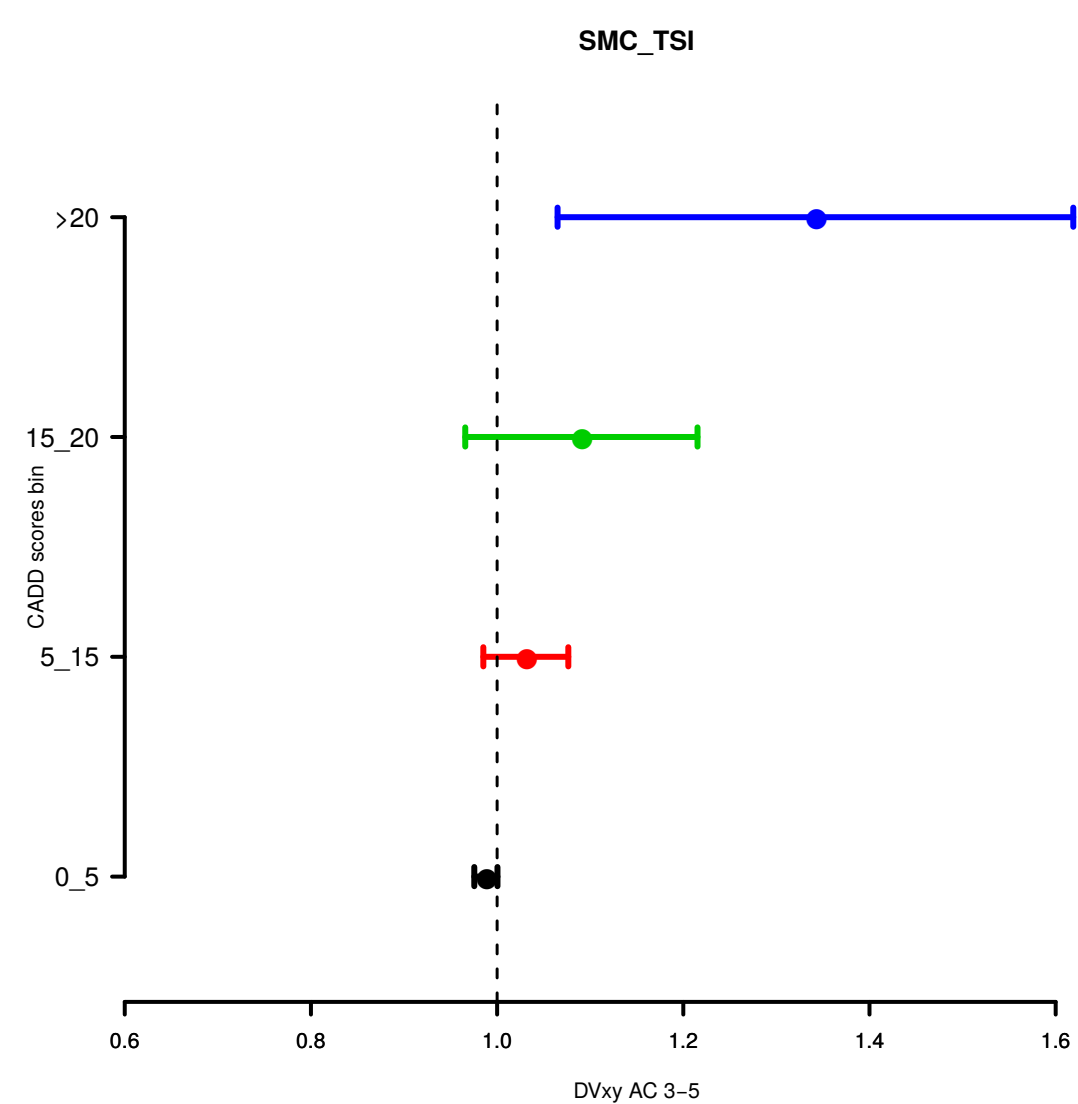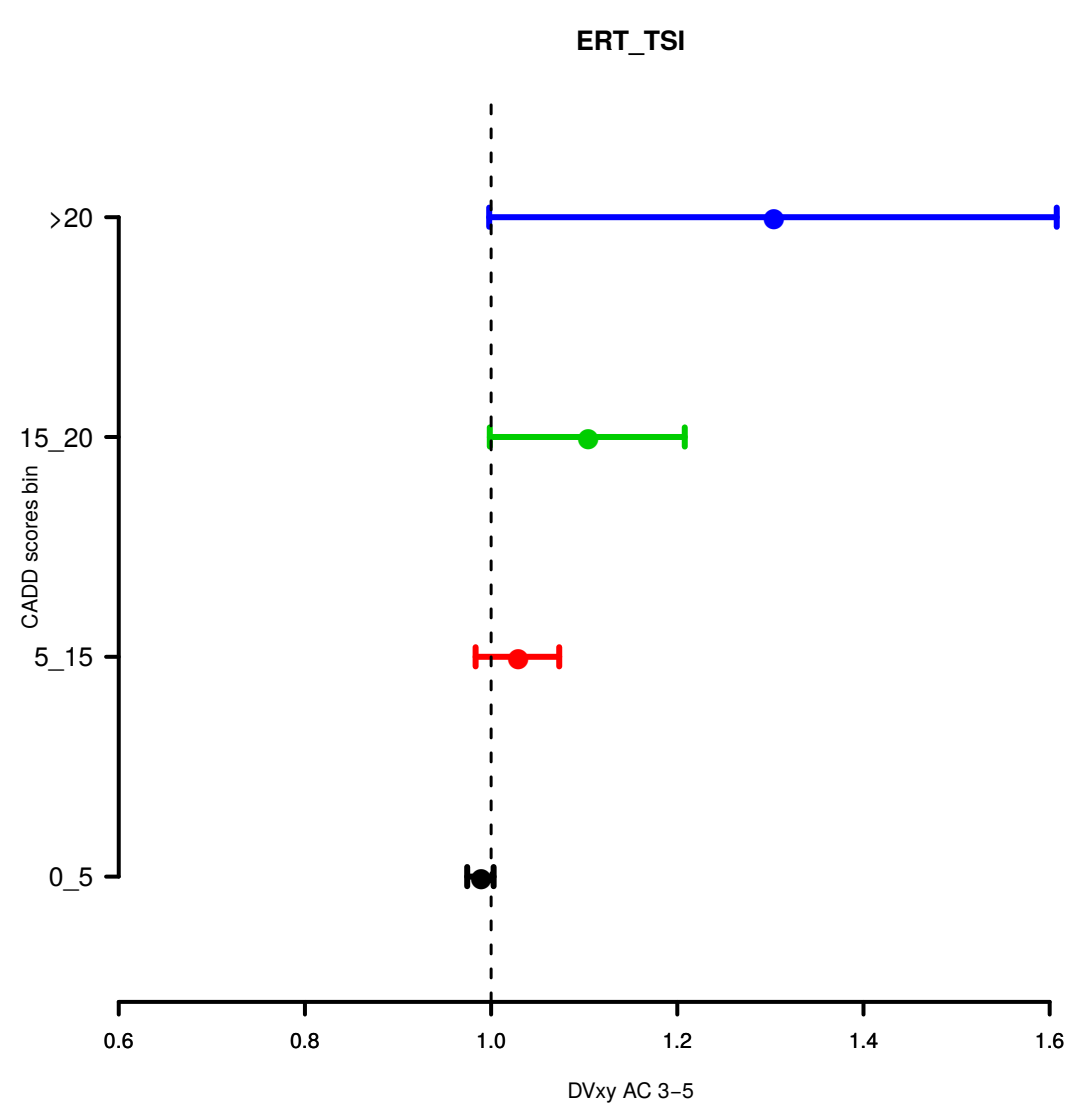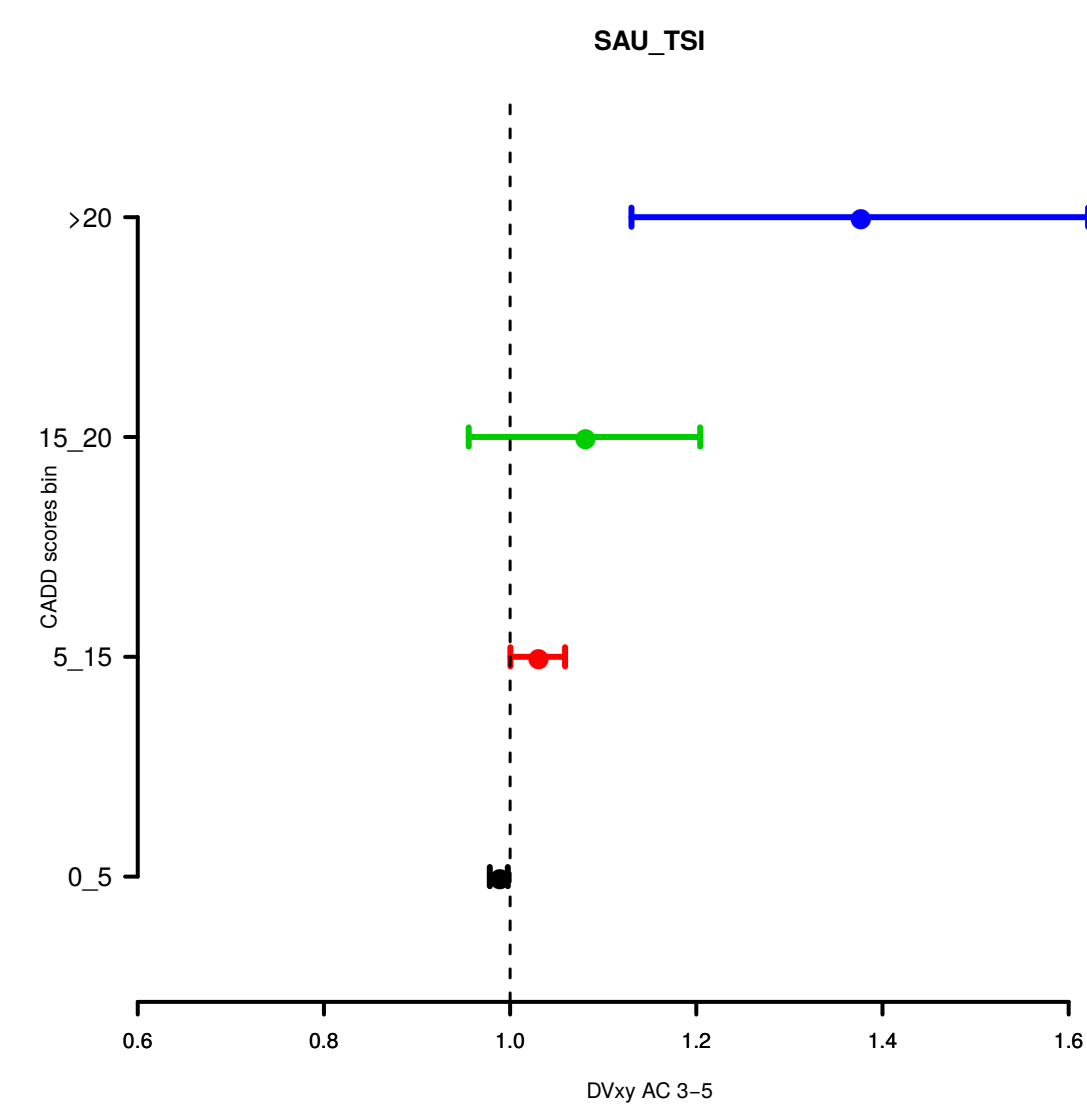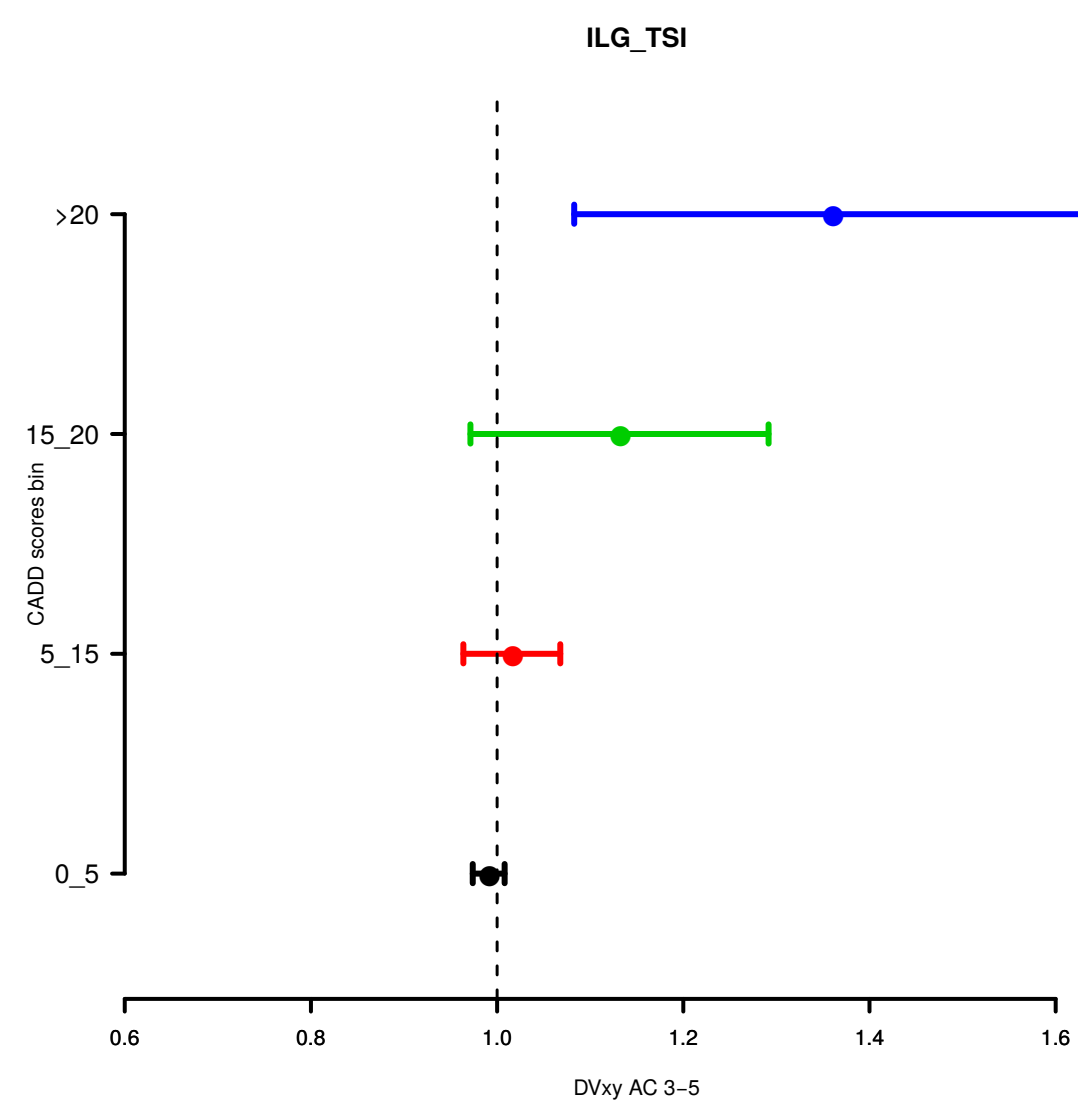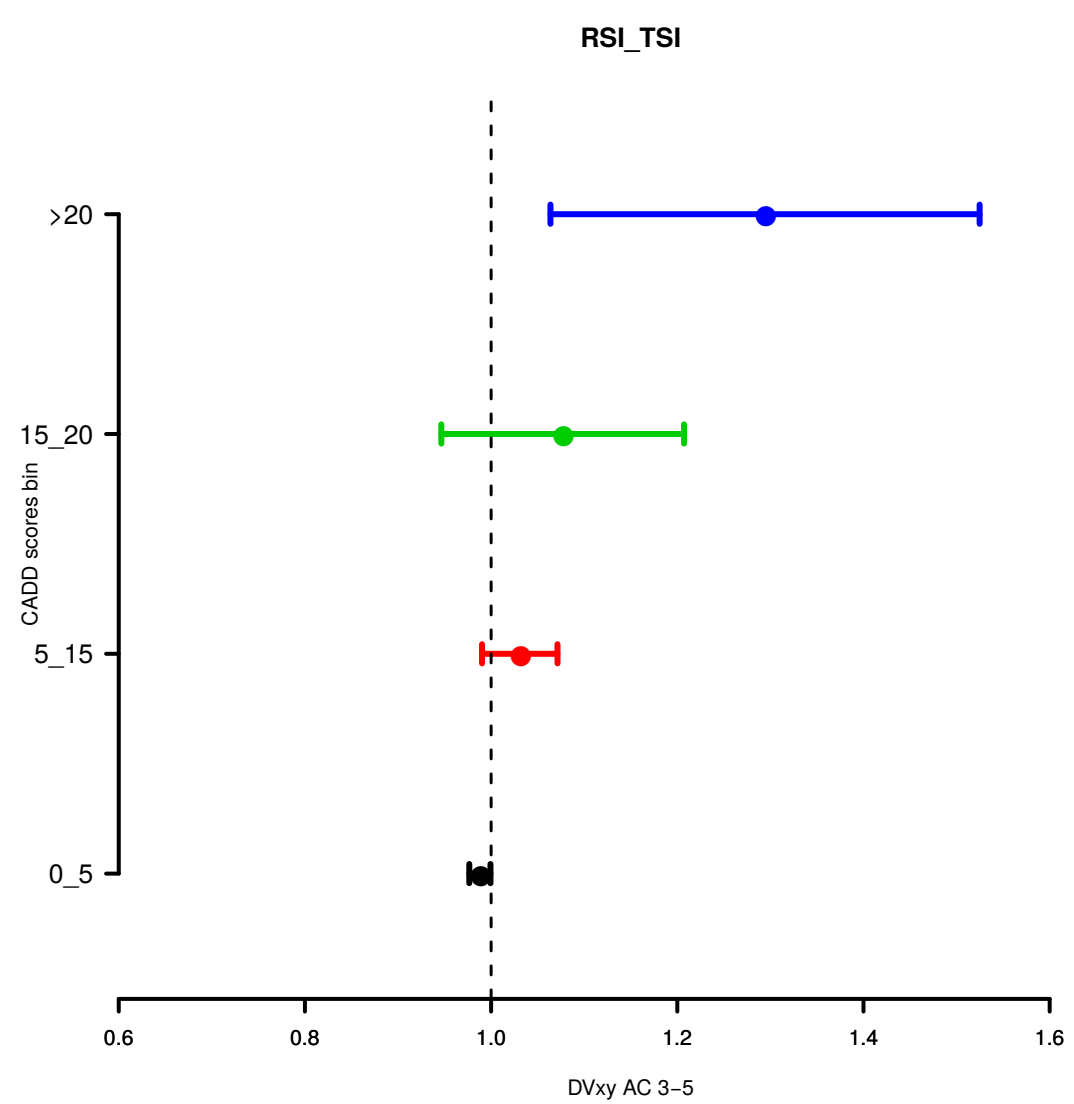

Supplement: Supplementary file 32 — Supplementary Figure 12 [file 41431_2019_551_MOESM32_ESM.pdf]

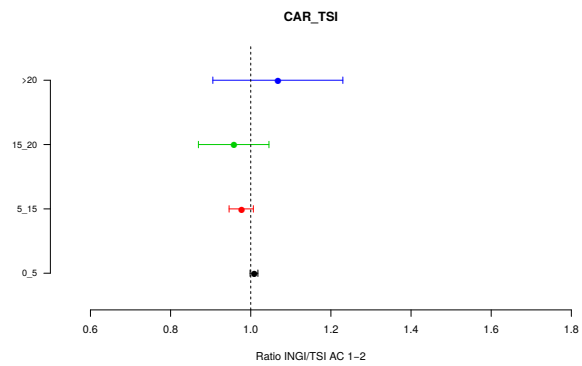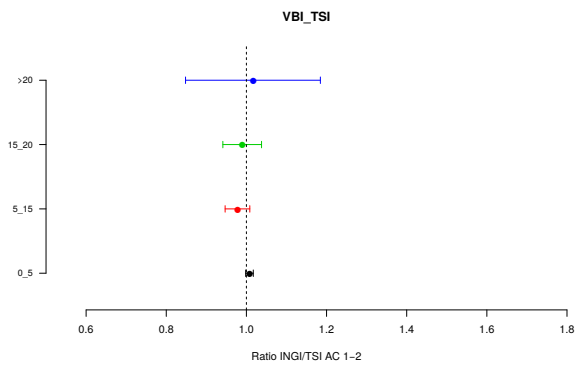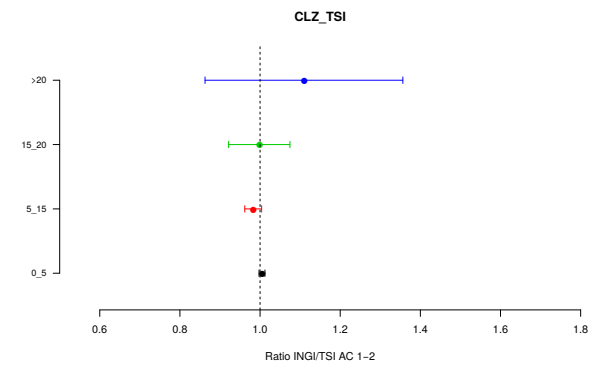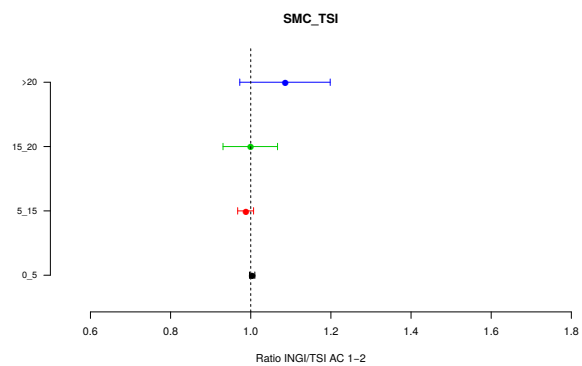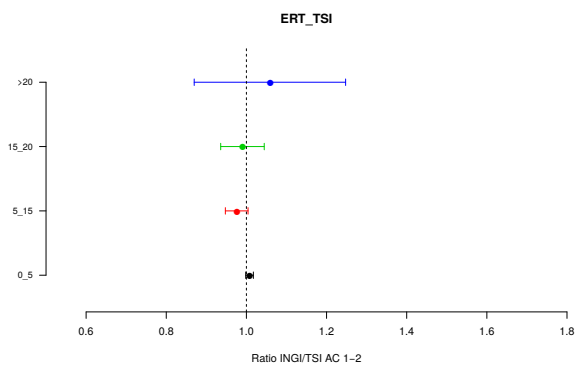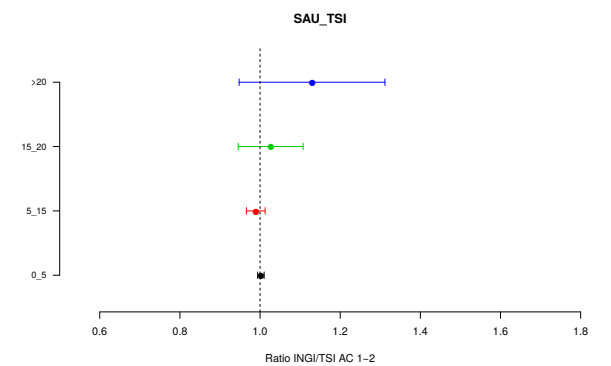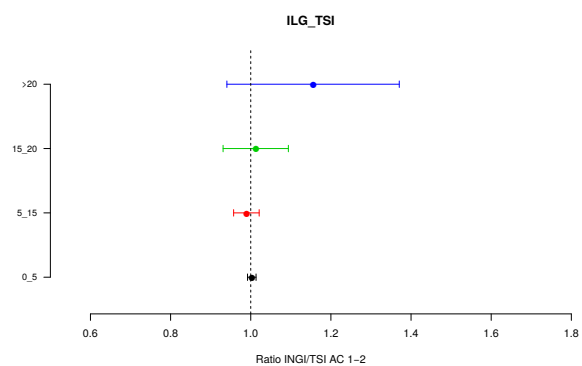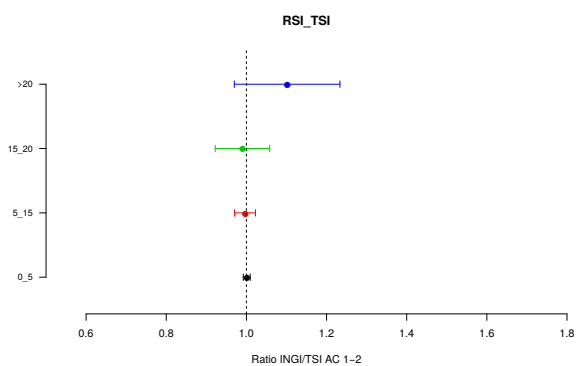

Supplementary Figure 13

Supplement: Supplementary file 33 — Supplementary Figure 13 [file 41431_2019_551_MOESM33_ESM.pdf]

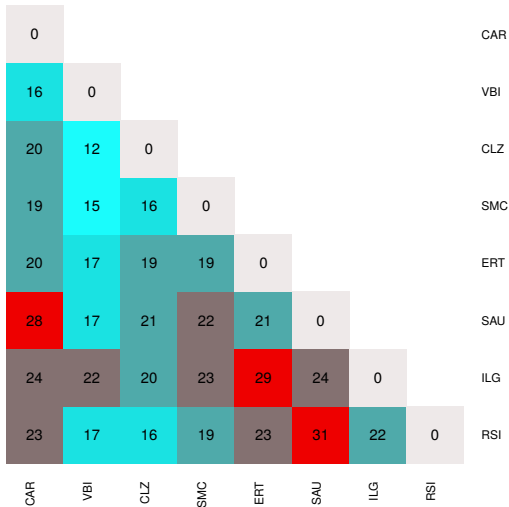

Supplementary Figure 14

Supplement: Supplementary file 34 — Supplementary Figure 14 [file 41431_2019_551_MOESM34_ESM.pdf]

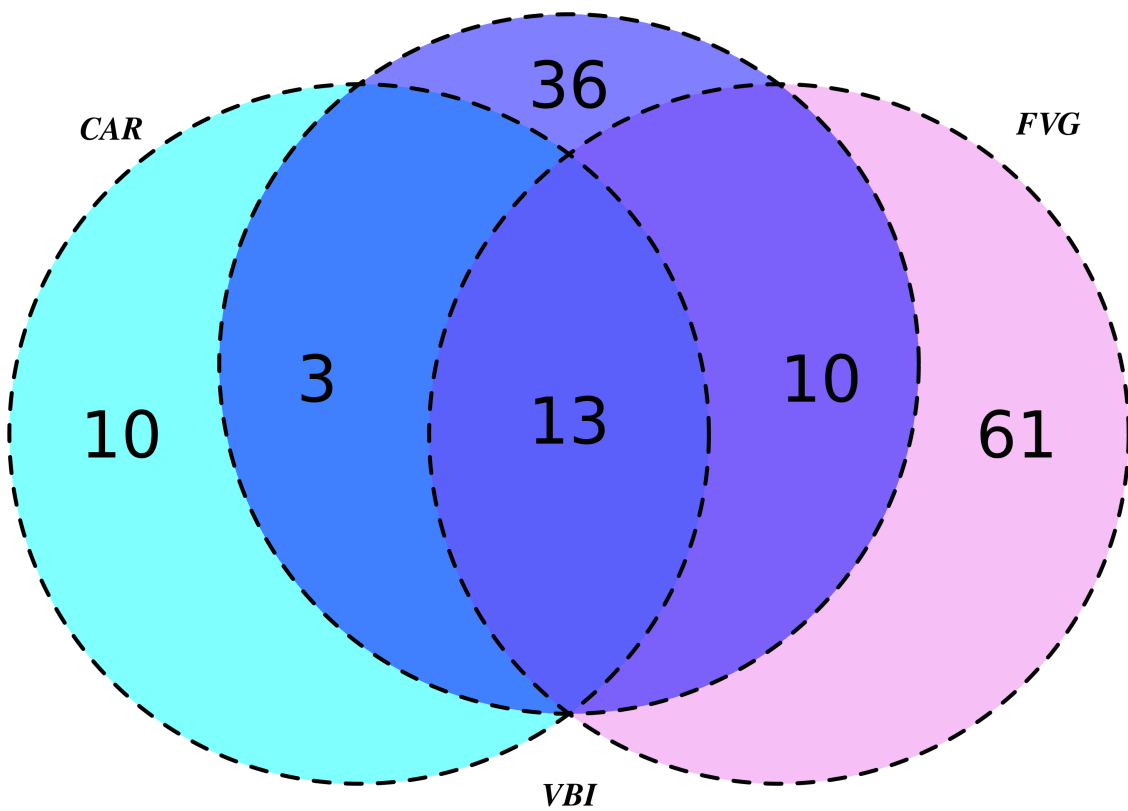

Supplementary Figure 15

Supplement: Supplementary file 35 — Supplementary Figure 15 [file 41431_2019_551_MOESM35_ESM.pdf]
